# Microbiota-mediated naringenin release limits postoperative acute gastrointestinal injury by suppressing ferroptosis

**DOI:** 10.1016/j.xcrm.2026.103101

**Published:** 2026-10-08

**Authors:** Fan Deng, Jingjuan Hu, Yue Min, Sun Xie, Zebin Lin, Qishun Sun, Huamin Liu, Shiying He, Siyuan Feng, Ling Wu, Xiaofeng Chen, Linghong Xu, Zheang Zhang, Wenqi Tang, Zhuang Li, Hongwei Zhou, Kexuan Liu

**Affiliations:** 1 Department of Anesthesiology, Nanfang Hospital, Southern Medical University, Guangzhou, Guangdong, China; 2 Guangdong Provincial Key Laboratory of Precision Anesthesia and Perioperative Organ Protection, Guangzhou, Guangdong, China; 3 Microbiome Medicine Center, Department of Laboratory Medicine, Zhujiang Hospital, Southern Medical University, Guangzhou, Guangdong, China; 4 Shenzhen Key Laboratory of Gastrointestinal Microbiota and Disease, Department of Gastroenterology, Gynecology and Obstetrics, and Laboratory Medicine, Shenzhen Hospital, Southern Medical University, Shenzhen, Guangdong, China; 5 State Key Laboratory of Multi-organ Injury Prevention and Treatment, Southern Medical University, Guangzhou, China

**Keywords:** acute gastrointestinal injury, cardiopulmonary bypass, gut microbiota, naringenin, ferroptosis

## Abstract

Acute gastrointestinal injury (AGI) remains a major complication after cardiopulmonary bypass, yet its microbiome-related determinants are unclear. We identify microbiota-mediated naringenin (NAR) release as a candidate modulator of AGI susceptibility. Patients who subsequently develop AGI have lower preoperative fecal NAR levels and β-glucuronidase (GUS) activity, accompanied by reduced abundance of *Bacteroides vulgatus* (*B. vulgatus*). In germ-free and conventional mice, *B. vulgatus* alleviates intestinal ischemia-reperfusion injury through GUS-dependent release of NAR from its glucuronide conjugate. Mechanistically, NAR engages the TLR4/MyD88-SPRR2A axis to suppress CHAC1-driven ferroptosis, thereby maintaining epithelial integrity and promoting mucosal repair *in vivo* and *in organoids*. Clinically, preoperative fecal NAR concentration and GUS activity show exploratory value as noninvasive markers for postoperative AGI risk stratification. Collectively, these findings support a microbiota-host metabolic model in which microbial NAR release may mitigate postoperative gastrointestinal injury by restricting ferroptosis, with potential implications for risk assessment and therapeutic modulation.

## Highlights

• Preoperative fecal naringenin and GUS activity are reduced in patients with AGI
• Microbial β-glucuronidase releases free naringenin from its glucuronide
• Naringenin suppresses epithelial ferroptosis via the TLR4/MyD88-SPRR2A axis
• Fecal naringenin and GUS support noninvasive AGI risk stratification

## Graphical abstract

**Figure:**
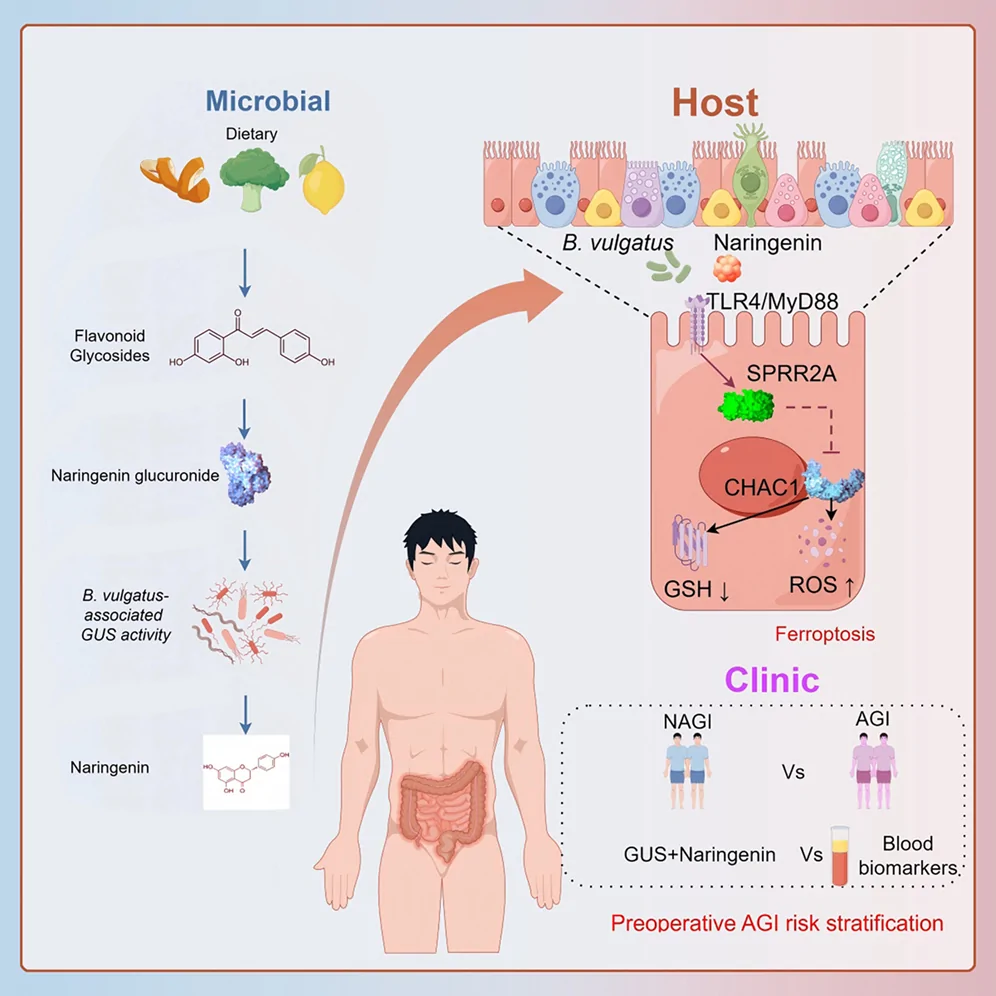

## Teaser

Deng et al. show that reduced preoperative fecal naringenin and β-glucuronidase activity associate with postoperative acute gastrointestinal injury after cardiopulmonary bypass. Mechanistically, microbial β-glucuronidase promotes naringenin release and limits epithelial ferroptosis, highlighting candidate noninvasive biomarkers and a potential microbiota-based intervention axis.

## Introduction

Acute gastrointestinal injury (AGI) represents a frequent and life-threatening complication after cardiopulmonary bypass (CPB) in cardiac surgery, exerting a substantial impact on postoperative morbidity and mortality.^1^^,^^2^ Despite improvements in perioperative management, AGI occurs in approximately 20% of patients undergoing cardiac surgery, and established cases are associated with mortality rates exceeding 50%.^3^^,^^4^ Early identification and prevention of AGI pose significant clinical challenges, as current risk stratification primarily relies on plasma biomarkers, such as diamine oxidase (DAO) and intestinal fatty acid-binding protein (I-FABP). These markers largely indicate advanced epithelial barrier disruption and exhibit limited sensitivity for detecting early, subclinical barrier compromise.^5^^,^^6^^,^^7^

Emerging evidence underscores the pivotal role of the gut microbiota in maintaining intestinal homeostasis and modulating host responses to critical illness and surgical stress.^8^^,^^9^^,^^10^ CPB-induced alterations in the gut microbiome have been implicated in the disruption of epithelial and immune barriers, promoting bacterial translocation and heightened secondary inflammatory responses that ultimately precipitate AGI.^11^^,^^12^ Beyond microbiota composition, microbial-derived metabolites have garnered attention as key mediators influencing epithelial integrity, redox homeostasis, and cell death pathways, particularly in the context of intestinal ischemia-reperfusion injury.^13^^,^^14^^,^^15^ However, the precise mechanisms through which specific commensal taxa and their metabolic activities facilitate the production of protective metabolites, and the contributory roles of these metabolites in mucosal repair, remain incompletely defined in the setting of postoperative AGI. Furthermore, whether distinctive microbial or metabolic profiles may serve as sensitive, non-invasive biomarkers for early AGI risk assessment is an important, unanswered clinical question.

To address these gaps, we systematically evaluated the mechanistic roles and biomarker potential of key commensal bacteria and their metabolites in CPB-associated AGI. Through integrated clinical cohort analyses and experimental models, we identified a *Bacteroides vulgatus* (*B. vulgatus*)-naringenin (NAR) axis that is associated with reduced AGI susceptibility and confers protection against intestinal injury in experimental models, in part by inducing SPRR2A-dependent suppression of CHAC1-mediated ferroptosis. These findings delineate a gut microbiota-metabolite-host interaction in AGI pathogenesis and support its potential value as an early warning biomarker and candidate perioperative intervention strategy.

## Results

### Microbiota-mediated NAR release is associated with postoperative AGI susceptibility after CPB

In a prospective cohort of 107 patients undergoing CPB, 21 patients (19.6%) developed postoperative AGI, revealing substantial interindividual variability in AGI susceptibility. Compared to patients without AGI (NAGI group, *n* = 86), AGI patients exhibited a more severe perioperative clinical course, including prolonged hospital and ICU stays, higher rates of enteral nutrition intolerance, and increased postoperative complications such as acute kidney injury and higher 3-month mortality (Figure 1A; Figures S1 and S2A–S2C; Table S1). Notably, baseline characteristics and intraoperative parameters, including age and surgical duration, were comparable between groups, suggesting that AGI susceptibility was not fully explained by measured clinical factors. Detailed perioperative data—including medications, nutritional support, and AGI onset time—are provided in Table S1. Together, these findings prompted us to investigate whether preoperative microbial and metabolic features were associated with subsequent AGI risk.

**Figure 1:**
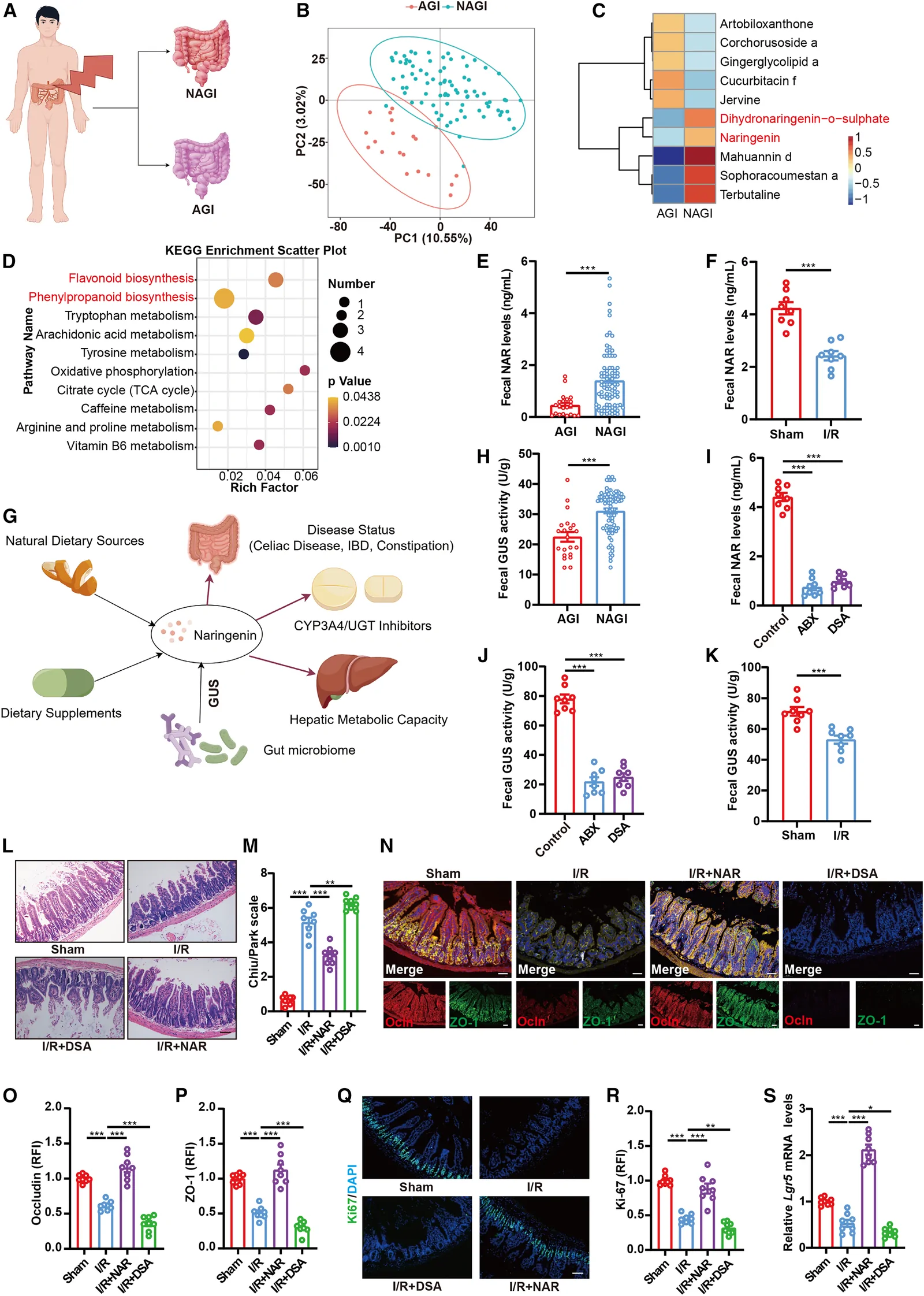
Gut microbiota-associated NAR is linked to postoperative AGI susceptibility after CPB (A) Patient stratification of CPB cohort (*n* = 107) into AGI, *n* = 21 and non-AGI (NAGI, *n* = 86) groups according to ESICM criteria. (B) PCoA of fecal metabolomic profiles in AGI and NAGI groups; statistical significance assessed by Adonis analysis. (C) Clustered heatmap showing differentially abundant fecal metabolites in preoperative samples from AGI and NAGI patients. (D) Bubble plot illustrating KEGG pathway enrichment of differential fecal metabolites between groups. (E and F) Quantitative LC-MS/MS measurement of NAR levels in fecal samples from AGI (*n* = 21) and NAGI (*n* = 86) patients (E) and from sham and intestinal I/R model mice (F) (*n* = 8 per group). (G) Schematic overview of NAR metabolic homeostasis. (H) Preoperative fecal β-glucuronidase (GUS) activity in AGI and NAGI patients. (I and J) Fecal NAR levels (I) and GUS activity (J) in ischemia/reperfusion (I/R) mice treated with vehicle, ABX, or D-saccharic acid-1,4-lactone (DSA, GUS inhibitor) (*n* = 8 per group). (K) Fecal GUS activity in sham and I/R mice (*n* = 8 per group). (L and M) Representative hematoxylin and eosin (H&E) staining (L) and pathological injury scores (M) of intestinal tissues (scale bars: 100 μm; *n* = 8 per group). (N–P) Immunofluorescence staining and quantitative analysis of ZO-1 and Occludin in intestinal sections (scale bars: 100 μm; *n* = 8 per group). (Q and R) Immunofluorescence staining and quantification of Ki67-positive cells in the intestine (scale bars: 100 μm; *n* = 8 per group). (S) Relative mRNA expression of *Lgr5* in intestinal tissue (*n* = 8 per group). Data are presented as mean ± SEM. Statistical significance was determined by Student’s *t* test (E, F, H, and K) or one-way ANOVA with Tukey’s post hoc test (I, J, and M–S). ∗*p* < 0.05, ∗∗*p* < 0.01, ∗∗∗*p* < 0.001. Each data point represents an individual biological replicate. Quantitative measurements were performed in triplicate. Abbreviations: ABX, antibiotics; AGI, acute gastrointestinal injury; GUS, β-glucuronidase; DSA, D-saccharic acid-1,4-lactone; H&E, hematoxylin and eosin; I/R, ischemia/reperfusion; NAR, naringenin; PCoA, principal coordinate analysis; RFI, relative fluorescence intensity.

To explore preoperative metabolic features associated with AGI, we profiled gut microbiota-related metabolites in fecal samples. Metabolomic analysis revealed that AGI patients had significantly lower levels of NAR and its metabolite dihydronaringenin-O-sulfate compared with NAGI patients (Figures 1B and 1C), with Kyoto Encyclopedia of Genes and Genomes (KEGG) pathway enrichment highlighting flavonoid and phenylpropanoid metabolism in the NAGI group (Figure 1D). Liquid chromatography-tandem mass spectrometry (LC-MS/MS) quantification confirmed reduced fecal NAR levels in patients with AGI. Because intestinal ischemia-reperfusion (I/R) is a major contributor to CPB-associated gut injury, we next examined a murine intestinal I/R model and observed similar reductions in fecal NAR (Figures 1E and 1F).

Analysis of factors governing NAR sources and metabolic fate suggested that fecal NAR levels were primarily linked to microbiota-dependent metabolism (Figure 1G; Table S2). Because microbial β-glucuronidase (GUS) catalyzes the deconjugation of NAR-glucuronide to release bioactive NAR, we next assessed fecal GUS activity. AGI patients exhibited significantly reduced preoperative fecal GUS activity (Figure 1H). In mice, both antibiotic-mediated microbiota depletion and GUS inhibition by D-saccharic acid-1,4-lactone (DSA) reduced fecal NAR levels and GUS activity, supporting a microbiota-GUS-NAR release axis (Figures 1I–1K).

Functionally, NAR supplementation protected against I/R-induced intestinal injury in mice by preserving mucosal barrier integrity and promoting epithelial regeneration, while GUS inhibition exacerbated tissue damage and impaired repair (Figures 1L–1S). Collectively, these findings indicate that reduced GUS-dependent microbial NAR release is associated with susceptibility to postoperative AGI after CPB and may functionally contribute to intestinal injury development.

### Preoperative gut microbiota transfers susceptibility to intestinal I/R injury

Preoperative 16S rRNA gene sequencing in CPB patients identified comparable α-diversity between AGI and NAGI groups, but β-diversity analysis revealed a pronounced separation of microbial communities (Figures S2D–S2F). Taxonomic profiling showed significant depletion of commensal *B. vulgatus* and enrichment of the pathobiont *Ruminococcus gnavus* in AGI patients (Figure 2A; Figure S2G). Integrated analysis demonstrated a strong positive correlation between *B. vulgatus* abundance and fecal NAR levels (r = 0.70, *p* = 0.02; Figure 2B), suggesting a link between commensal depletion and impaired microbial NAR release.

**Figure 2:**
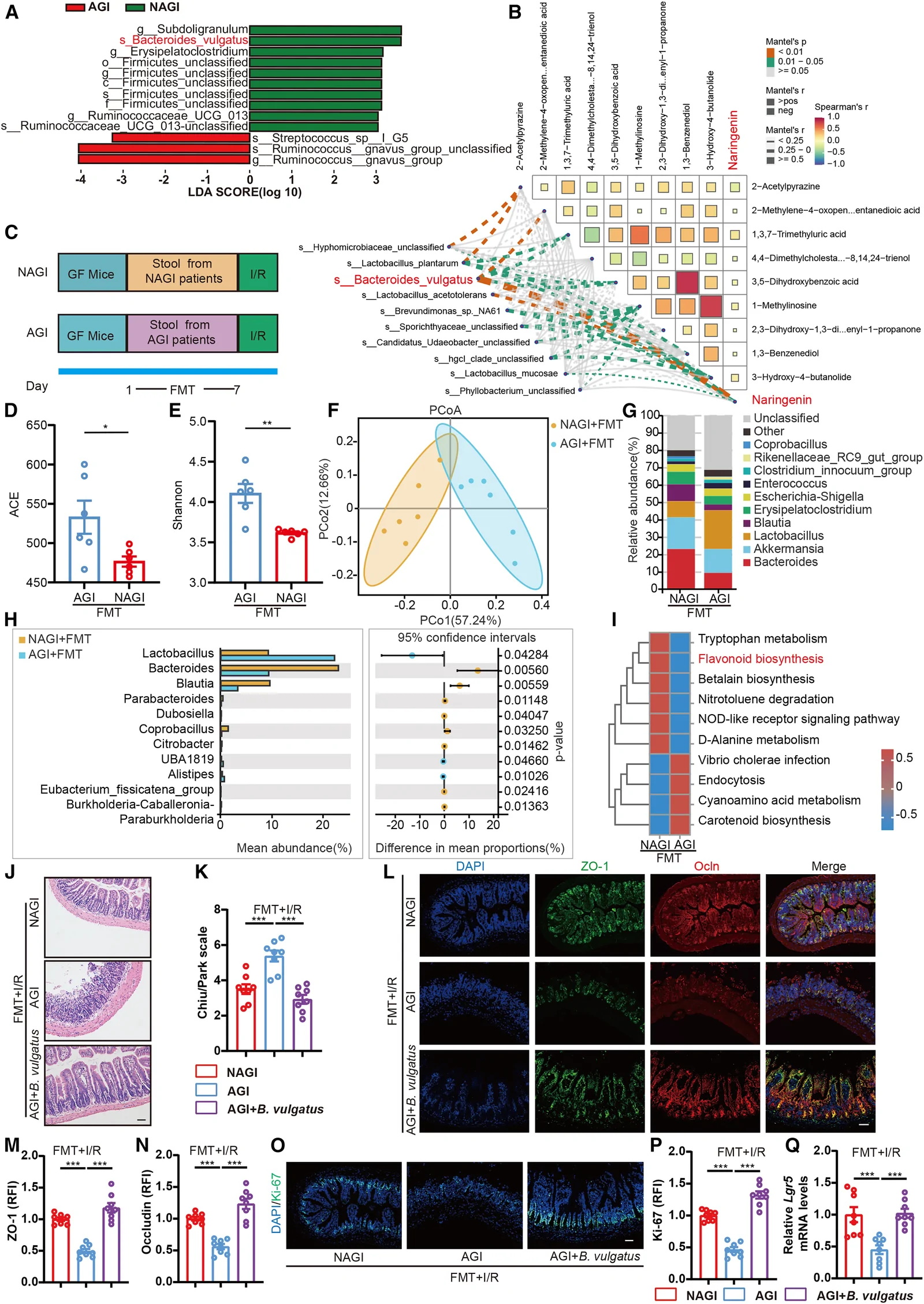
Gut microbiota from patients with AGI transfers susceptibility to intestinal I/R injury (A) Differential gut microbiota signatures between patients with acute gastrointestinal injury (AGI, *n* = 21) and non-AGI (NAGI, *n* = 86) identified by linear discriminant analysis effect size (LEfSe). (B) Spearman correlations between fecal microbial species and metabolites in AGI and NAGI patients. (C) Schematic representation of FMT from AGI and NAGI patient donors into GF mice. (D and E) Alpha diversity indices in AGI-FMT and NAGI-FMT recipient mice (*n* = 6 per group). (F) PCoA of microbial β-diversity between AGI-FMT and NAGI-FMT groups (*n* = 6 per group). (G) Taxonomic composition of fecal microbiota at the genus level in FMT recipient mice (*n* = 6 per group). (H) Differential abundance analysis of indicator genera between AGI-FMT and NAGI-FMT groups (*n* = 6 per group). (I) KEGG pathway prediction of the gut microbiome in FMT recipient mice (*n* = 6 per group). (J–Q) Assessment of intestinal injury and repair following I/R (*n* = 8 per group, scale bars: 100 μm): (J and K) Representative H&E staining and quantification of injury scores; (L–N) Immunofluorescence staining and quantitative analysis of ZO-1 and Occludin; (O and P) Immunofluorescence detection and quantification of Ki67-positive cells; (Q) Relative expression of *Lgr5* mRNA in intestinal tissue. Data are presented as mean ± SEM. LEfSe was used for differential taxonomic analysis (A). Statistical significance was assessed by Spearman correlation (B), Welch’s *t* test (D and E), Adonis analysis (F), and one-way ANOVA with Tukey’s post hoc test (K–Q). ∗*p* < 0.05, ∗∗*p* < 0.01, ∗∗∗*p* < 0.001. Each data point represents an individual biological replicate. Quantitative measurements were performed in triplicate. Abbreviations: AGI, acute gastrointestinal injury; FMT, fecal microbiota transplantation; GF, germ-free.

To test whether preoperative microbiota could functionally transfer injury susceptibility, we transplanted fecal microbiota from preoperative samples of AGI or NAGI donors into germ-free (GF) mice (Figure 2C). NAGI-fecal microbiota transplantation (FMT) recipients recapitulated key features of donor microbiota, including greater *Bacteroides* representation and enrichment of flavonoid biosynthetic pathways (Figures 2D–2I). After I/R challenge, NAGI-FMT mice developed less mucosal injury, higher Occludin and ZO-1 expression, increased epithelial proliferation, and greater *Lgr5*^+^ stem cell abundance than AGI-FMT mice (Figures 2J–2Q). Notably, supplementation of AGI-FMT mice with *B. vulgatus*, which exhibited high GUS activity, largely attenuated the injury phenotype.

Together, these findings support a functional contribution of the preoperative microbiota and commensal-associated metabolic activity to intestinal I/R injury susceptibility in recipient mice.

### *B. vulgatus* protects against intestinal I/R injury through GUS-dependent release of NAR

Given the association between *B. vulgatus* abundance, GUS activity, and fecal NAR levels in CPB patients, we investigated whether *B. vulgatus* contributes to protection against intestinal I/R injury through this metabolic axis. Mono-colonization of GF mice with *B. vulgatus* prior to I/R challenge significantly mitigated mucosal damage, improved barrier integrity (elevated Occludin and ZO-1), and enhanced epithelial repair (increased Ki67^+^ and Lgr5^+^ cells) compared to controls (Figures 3A and 3B; Figures S3A–S3F). In conventional mice, only live—not heat-killed—*B. vulgatus* enriched the *Bacteroidaceae* family (Figures 3C and 3D; Figures S3G–S3J), increased intestinal GUS activity (Figure 3E), and conferred comparable protection, as shown by reduced pathology and improved repair markers (Figures 3F and 3G; Figures S3K–S3P). Consistently, fecal metabolomics showed that live *B. vulgatus* restored NAR levels post-I/R (Figure 3H), prompting us to investigate the underlying mechanism.

**Figure 3:**
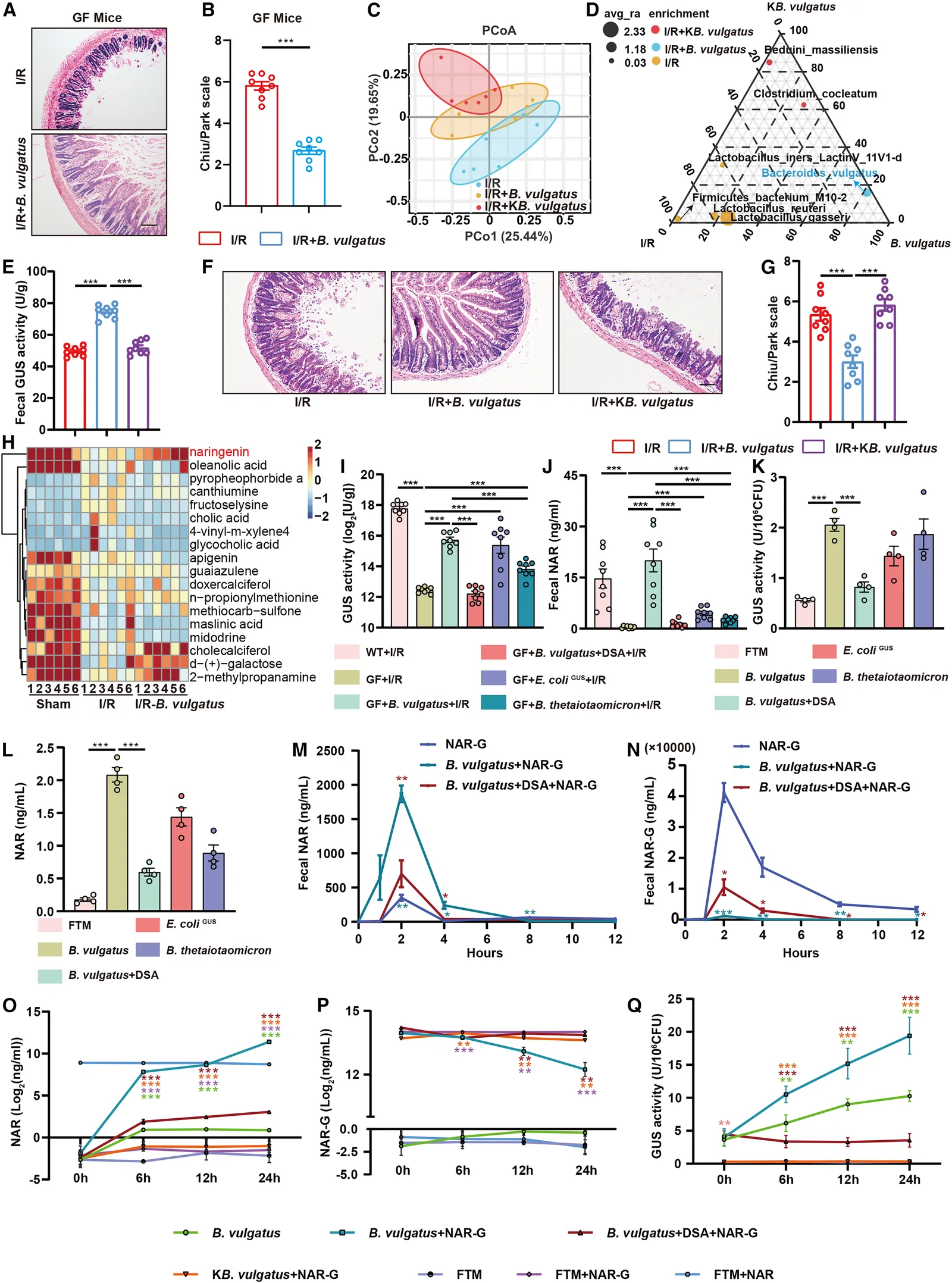
*B. vulgatus* protects against intestinal I/R injury through GUS-dependent NAR release (A and B) Representative H&E staining (A) and quantification of pathological damage scores (B) in intestinal tissues from germ-free (GF) mice subjected to I/R or I/R with *B. vulgatus* administration (I/R + *B. vulgatus*) (scale bars: 100 μm; *n* = 8 per group). (C) PCoA of microbial β-diversity among I/R, I/R + *B. vulgatus*, and I/R + heat-killed *B. vulgatus* (K *B. vulgatus*) groups (*n* = 6 per group). (D) Three-dimensional heatmap displaying gut microbiota composition across I/R, I/R + *B. vulgatus*, and I/R + K. *B. vulgatus* groups (*n* = 6 per group). (E) Fecal GUS activity in mice (*n* = 8 per group). (F and G) H&E staining (F) and pathological injury scores (G) of intestinal tissues from I/R, I/R + *B. vulgatus*, and I/R + K. *B. vulgatus* groups (scale bars: 100 μm; *n* = 8 per group). (H) Heatmap of differentially abundant cecal metabolites among sham, I/R, and I/R + *B. vulgatus* groups (*n* = 6 per group). (I and J) Fecal GUS activity and NAR levels in GF mice (*n* = 8 per group). (K) *In vitro* GUS activity of bacterial cultures (*n* = 4 per group). (L) NAR concentrations in bacterial culture supernatants (*n* = 4 per group). (M) Temporal changes in fecal NAR concentration (*n* = 3 per group). (N) Temporal changes in fecal NAR-G concentration (*n* = 4 per group). (O–Q) Temporal changes in NAR concentrations (O), NAR-G concentrations (P), and GUS activity (Q) in bacterial cultures grown in fluid thioglycollate medium (FTM) (*n* = 4 per group). Data are presented as mean ± SEM. Statistical analysis was performed by Welch’s *t* test (B) and one-way ANOVA with Tukey’s post hoc test for multiple comparisons. ∗*p* < 0.05, ∗∗*p* < 0.01, ∗∗∗*p* < 0.001. The asterisks of different colors (∗, ∗∗, ∗∗∗) in the figure are used to indicate the statistical significance of intergroup comparisons, with the color of the asterisks matching the color of the corresponding group. Each data point represents an individual biological replicate. Quantitative measurements were performed in triplicate. Abbreviations: GUS, β-glucuronidase; *B. vulgatus*, *Bacteroides vulgatus*; FTM, fluid thioglycollate medium; GF, germ-free; K. *B. vulgatus*, heat-killed *Bacteroides vulgatus*; NAR, naringenin; NAR-G, naringenin-7-O-glucuronide.

We next examined whether the protective effect of *B. vulgatus* was associated with its GUS activity. Mono-colonization with another GUS-positive commensal, *B. thetaiotaomicron,* conferred significantly weaker protection than *B. vulgatus* across all parameters (Figures S4A–S4G). Importantly, GUS inhibition by DSA abrogated the protection in *B. vulgatus*-colonized mice, whereas GUS-overexpressing *E. coli* partially phenocopied this effect (Figures S4A–S4G). Accordingly, *B. vulgatus*, *B. thetaiotaomicron*, and GUS-overexpressing *E. coli* each increased fecal GUS activity and NAR levels *in vivo* (Figures 3I and 3J), with *B. vulgatus* inducing significantly greater increases than *B. thetaiotaomicron*; DSA suppressed both to near-background levels. Corresponding patterns were observed *in vitro* for bacterial GUS activity and supernatant NAR levels (Figures 3K and 3L).

NAR is typically present *in vivo* predominantly as phase II conjugates (e.g., naringenin-7-O-glucuronide, NAR-G), and gut microbial GUS can regenerate bioactive free NAR via deconjugation. We next asked whether *B. vulgatus* could convert the conjugated precursor NAR-G into free NAR. *In vivo*, colonization with *B. vulgatus* significantly enhanced fecal availability of free NAR after oral NAR-G administration (Figures 3M and 3N), as reflected by higher fecal Cmax (Figure S4H) and area under the curve (AUC_0–12 h_) (Figure S4I), an increased fecal NAR/NAR-G ratio (Figure S4J), and elevated GUS activity (Figure S4K), effects that were largely reversed by DSA. *In vitro*, only live *B. vulgatus* efficiently hydrolyzed NAR-G to release free NAR; this process was abolished by heat inactivation or DSA (Figures 3O–3Q). To test whether NAR could contribute to epithelial protection, we employed an intestinal organoid hypoxia/reoxygenation (H/R) injury model. Both *B. vulgatus* supernatant and exogenous NAR enhanced organoid viability (Figures S5A and S5B), barrier function (Figures S5C–S5E), proliferation (Figures S5F and S5G), and *Lgr5* expression (Figure S5H). Together, these findings identify a GUS-dependent, *B. vulgatus*-associated NAR release pathway that enhances epithelial resilience to intestinal I/R injury.

### NAR engages the TLR4/MyD88-SPRR2A pathway to suppress CHAC1 and protect against intestinal I/R injury

To clarify how *B. vulgatus* and the metabolite NAR confer intestinal protection, we performed transcriptomic profiling of I/R-injured mouse intestines treated with NAR or *B. vulgatus*. Principal component analysis (PCA) revealed distinct transcriptional signatures among untreated, NAR-treated, and bacteria-treated cohorts (Figure S6A). Differential expression analysis showed that I/R-induced *Sprr2a* downregulation and *Chac1* upregulation were both reversed by NAR or *B. vulgatus* treatment (Figure 4A; Figures S6B and S6C). Notably, *Sprr2a* and *Chac1* levels were strongly inversely correlated (r = −0.813, *p* < 0.001; Figure 4B), suggesting regulatory interplay. Molecular docking and SPR assays supported a direct interaction between SPRR2A and CHAC1 (Figures 4C and 4D). To test the functional relevance of this axis, we examined *Sprr2a*^−/−^ mice after I/R injury. *Sprr2a* deficiency increased epithelial CHAC1 expression, exacerbated intestinal injury, and largely abolished the CHAC1-suppressive and epithelial-protective effects of *B. vulgatus* or NAR (Figures 4E–4H; Figures S6D–S6I). These findings identify SPRR2A as a key downstream effector linking the *B. vulgatus*–NAR axis to CHAC1 suppression and epithelial protection.

**Figure 4:**
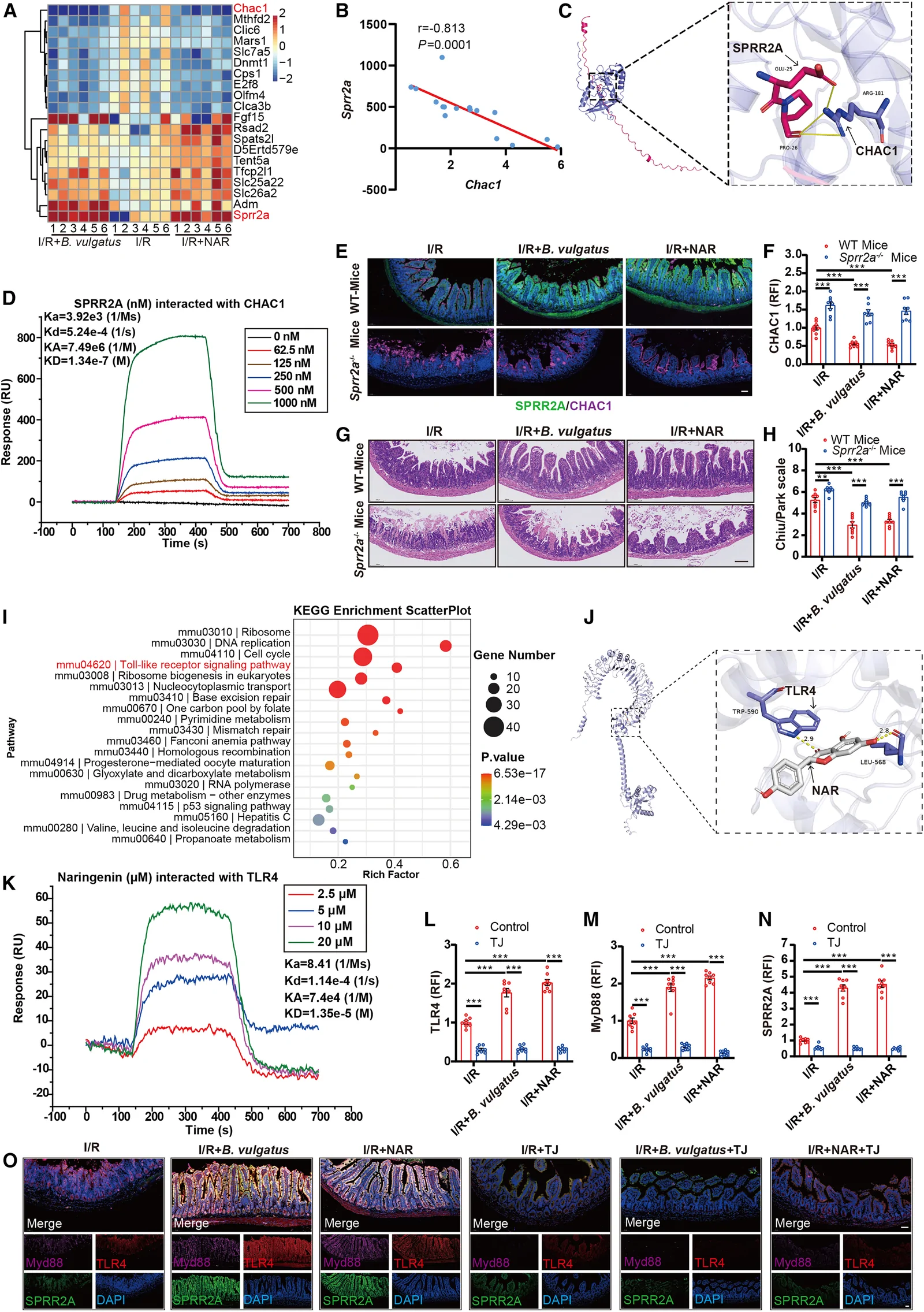
NAR engages the TLR4/MyD88-SPRR2A pathway to suppress CHAC1 and protect against intestinal I/R injury (A) Heatmap showing differential gene expression profiles among intestinal tissues from I/R, I/R + *B. vulgatus*, and I/R + NAR treated mice (*n* = 6 per group). (B) Correlation analysis between *Sprr2a* and *Chac1* expression levels. (C) Molecular docking simulation of the SPRR2A–CHAC1 interaction. (D) Surface plasmon resonance (SPR) analysis of SPRR2A (0–1,000 nM) binding to recombinant human (rh) CHAC1 protein. (E) Immunofluorescence staining of SPRR2A and CHAC1 in intestinal tissues from wild-type (WT) and *Sprr2a*^−/−^ mice after I/R, I/R + *B. vulgatus*, or I/R + NAR treatment (scale bars: 100 μm; *n* = 8 per group). (F) Quantitative analysis of CHAC1 expression in intestinal tissues (*n* = 8 per group). (G and H) Representative H&E staining (G) and pathological damage scores (H) in intestinal tissues from WT and *Sprr2a*^−/−^ mice subjected to I/R, I/R + *B. vulgatus*, or I/R + NAR (scale bars: 100 μm; *n* = 8 per group). (I) KEGG enrichment analysis of transcriptomic data highlighting TLR4 signaling pathway enrichment. (J) Computational molecular docking of NAR with the TLR4 protein (PDB: 3FXI). (K) SPR analysis of NAR (2.5–20 μM) binding to rhTLR4 (Sf9, His, GST tagged). (L–O) Immunofluorescence staining and quantification of TLR4, MyD88, and SPRR2A in intestinal tissues from WT mice treated with or without the MyD88 inhibitor TJ-M2010-5 after I/R, I/R + *B. vulgatus*, or I/R + NAR treatment (scale bars: 100 μm; *n* = 8 per group). Data are presented as mean ± SEM. Statistical analysis was performed using Spearman correlation (B) and two-way ANOVA with Tukey’s post hoc test. ∗*p* < 0.05, ∗∗*p* < 0.01, ∗∗∗*p* < 0.001. Each data point represents an individual biological replicate. Quantitative measurements were performed in triplicate. Abbreviations: CHAC1, ChaC glutathione-specific γ-glutamylcyclotransferase 1; SPRR2A, small proline-rich protein 2A; SPR, surface plasmon resonance; WT, wild-type.

To elucidate the upstream mechanism, KEGG pathway analysis of transcriptomes implicated the TLR4 signaling pathway in the response to NAR or *B. vulgatus* treatment (Figure 4I). Correspondingly, both interventions promoted TLR4/MyD88 signaling activation (Figures S6J and S6K). Molecular docking and surface plasmon resonance (SPR) assays further supported a direct interaction between NAR and the TLR4 complex (Figures 4J and 4K). Pharmacological inhibition of TLR4/MyD88 signaling (TJ-M2010-5) largely blocked *B. vulgatus*/NAR-induced TLR4/MyD88 activation and downstream SPRR2A upregulation (Figures 4L–4O).

Taken together, these results support a mechanistic pathway in which NAR engages TLR4/MyD88 signaling, leading to SPRR2A upregulation, CHAC1 suppression, and enhanced intestinal barrier defense following I/R injury.

### *B. vulgatus* and NAR protect against intestinal I/R injury through CHAC1-dependent suppression of ferroptosis

CHAC1 has been implicated in ferroptosis through its role in promoting glutathione depletion and oxidative stress. KEGG pathway enrichment analysis of fecal metabolites revealed significant enrichment of ferroptosis-related pathways in I/R mice relative to *B. vulgatus*-colonized mice (Figure 5A), implicating ferroptosis as a potential contributor to intestinal injury.

**Figure 5:**
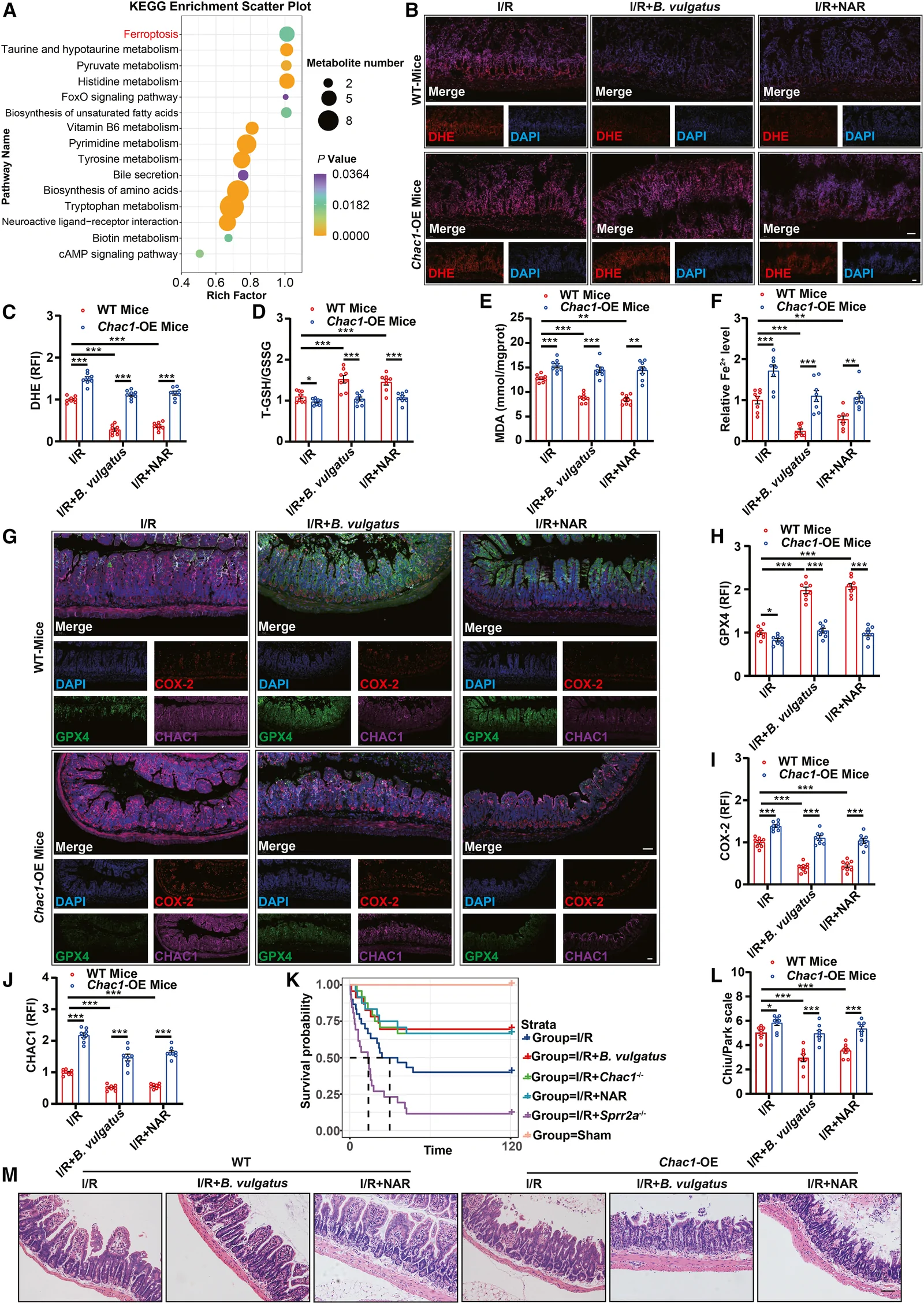
*B. vulgatus* and NAR protect against intestinal I/R injury by suppressing CHAC1-mediated ferroptosis in mice (A–C) (A) Functional prediction analysis of fecal metabolomics in intestinal I/R and *B. vulgatus*-colonized mice (*n* = 6 per group). (B and C) DHE staining (B) and quantitative analysis (C) of intestinal oxidative stress in WT or *Chac1*-overexpressing (*Chac1*-OE) mice subjected to I/R, I/R + *B. vulgatus*, or I/R + naringenin (NAR) treatment (scale bars: 100 μm; *n* = 8 per group). (D–F) Quantification of T-GSH/GSSG ratio (D), MDA (malondialdehyde) (E), and Fe^2+^ (F) content in intestinal tissues from WT and *Chac1*-OE mice (*n* = 8 per group). (G) Multiplex immunofluorescence staining of GPX4, COX-2, and CHAC1 in intestinal tissues from WT or *Chac1*-OE mice after I/R, I/R + *B. vulgatus*, or I/R + NAR treatment (scale bars: 100 μm; *n* = 8 per group). (H–J) Relative fluorescence intensity quantification of GPX4 (H), COX-2 (I), and CHAC1 (J) in intestinal tissues from WT and *Chac1*-OE mice (*n* = 8 per group). (K) Five-day survival analysis in WT mice (sham, I/R, I/R + *B. vulgatus*, I/R + NAR), in *Sprr2a*^−/−^ mice (I/R), and in *Chac1*^−/−^ mice (I/R) (*n* = 15 per group). (L and M) Representative H&E staining (L) and pathological damage scores (M) of intestinal tissues from WT or *Chac1*-OE mice under indicated treatments (scale bars: 100 μm; *n* = 8 per group). Data are presented as mean ± SEM. Statistical analysis was performed by log rank test (K) and two-way ANOVA followed by Tukey’s post hoc test. ∗*p* < 0.05, ∗∗*p* < 0.01, ∗∗∗*p* < 0.001. Each data point represents an individual biological replicate. Quantitative measurements were performed in triplicate. Abbreviations: DHE, dihydroethidium; GPX4, glutathione peroxidase 4; COX-2, cyclooxygenase-2; CHAC1, ChaC glutathione-specific γ-glutamylcyclotransferase 1; MDA, malondialdehyde; T-GSH/GSSG, total glutathione-to-oxidized glutathione ratio.

In wild-type (WT) mice, both *B. vulgatus* colonization and NAR administration attenuated I/R-induced ferroptosis, as shown by reduced ROS accumulation (Figures 5B and 5C), restored the T-GSH/GSSG ratio (Figure 5D), decreased MDA content (Figure 5E), reduced Fe^2+^ accumulation (Figure 5F), increased GPX4, and decreased COX-2 and CHAC1 expression (Figures 5G–5J). Survival analysis further showed that *B. vulgatus* colonization, NAR administration, or *Chac1* knockout improved survival after I/R, whereas *Sprr2a* deficiency reduced survival (Figure 5K). To test whether CHAC1 suppression contributed to these effects, we induced epithelial *Chac1* overexpression (*Chac1*-OE) *in vivo*. *Chac1* overexpression largely abolished the anti-ferroptosis effects of *B. vulgatus* or NAR (Figures 5B–5J) and blunted their epithelial-protective effects, including histological preservation (Figures 5L and 5M), restoration of Occludin and ZO-1 expression, epithelial proliferation, and *Lgr5* expression (Figures S7A–S7F). These *in vivo* data support a role for CHAC1 in modulating ferroptosis-associated epithelial injury after I/R.

We next examined this mechanism in an intestinal organoid H/R injury model. In WT organoids, pretreatment with *B. vulgatus* supernatant or NAR attenuated ferroptosis-associated injury, as shown by reduced ROS^+^ cell accumulation (Figure 6A), normalization of the T-GSH/GSSG ratio (Figure 6B), diminished lipid peroxidation (Figure 6C), decreased intracellular Fe^2+^ (Figure 6D), upregulated GPX4, and suppressed COX-2 and CHAC1 (Figures 6E–6H). These effects were largely lost in *Chac1*-OE organoids (Figures 6A–6H). Consistently, *B. vulgatus* supernatant and NAR improved organoid viability and preserved organoid morphology (Figures 6I and 6J), restored Occludin and ZO-1 expression (Figures S7G–S7I), increased epithelial proliferation (Figures S7J and S7K), and enhanced *Lgr5* expression (Figure S7L), whereas *Chac1* overexpression largely reversed these protective effects (Figures 6I and 6J; Figures S7G–S7L).

**Figure 6:**
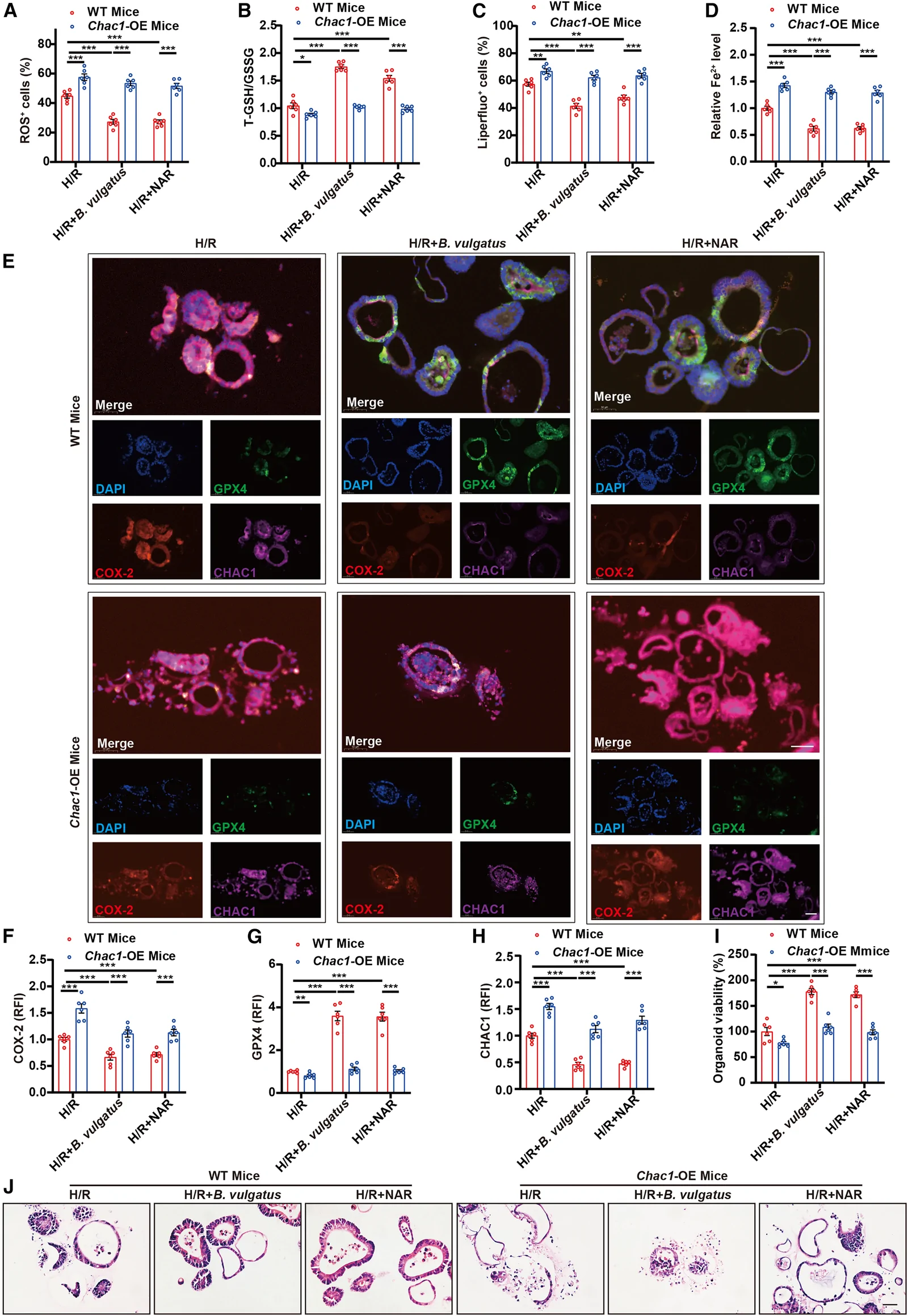
*B. vulgatus* and NAR suppress CHAC1-mediated ferroptosis in intestinal organoids (A) Measurement of reactive oxygen species (ROS) levels in intestinal organoids derived from wild-type (WT) and *Chac1*-overexpressing (*Chac1*-OE) mice following H/R, H/R + *B. vulgatus*, or H/R + NAR treatments (*n* = 6 per group). (B–D) Quantification of T-GSH/GSSG ratio (B), Liperfluo signal intensity (lipid peroxidation marker, C), and Fe^2+^ levels (D) in intestinal organoids from WT and *Chac1*-OE mice (*n* = 6 per group). (E–H) Multiplex immunofluorescence staining and quantitative analysis of GPX4, COX-2, and CHAC1 expression in intestinal organoids derived from WT and Chac1-OE mice (scale bars: 50 μm; *n* = 6 per group). (I) Organoid viability across indicated groups (*n* = 6 per group). (J) Representative H&E staining of intestinal organoids derived from WT and *Chac1*-OE mice (scale bars: 50 μm; *n* = 6 per group). Data are presented as mean ± SEM. Statistical analysis was performed by two-way ANOVA with Tukey’s post hoc test. ∗*p* < 0.05, ∗∗*p* < 0.01, ∗∗∗*p* < 0.001. Each data point represents an individual biological replicate. Quantitative measurements were performed in triplicate. Abbreviations: GPX4, glutathione peroxidase 4; COX-2, cyclooxygenase-2; CHAC1, ChaC glutathione-specific γ-glutamylcyclotransferase 1; T-GSH/GSSG, total glutathione-to-oxidized glutathione ratio; NAR, naringenin; H/R, hypoxia/reoxygenation.

Together, these findings indicate that CHAC1 suppression is an important component of the *B. vulgatus*-NAR protective axis, linking ferroptosis control to epithelial preservation and barrier repair after intestinal I/R injury.

### Preoperative fecal GUS activity and NAR predict postoperative AGI risk after CPB

Dynamic profiling of perioperative plasma AGI biomarkers in the modeling cohort showed that DAO, D-lactate, and IFABP were significantly elevated only in patients who developed AGI at 6 h postoperatively (T3), whereas no significant intergroup differences were observed preoperatively (T1), at the end of operation (T2), or 24 h postoperatively (T4) (Figures 7A–7C). These findings suggest that early barrier dysfunction may accompany AGI development, although the utility of plasma markers is limited by their postoperative timing and restricted temporal window.

**Figure 7:**
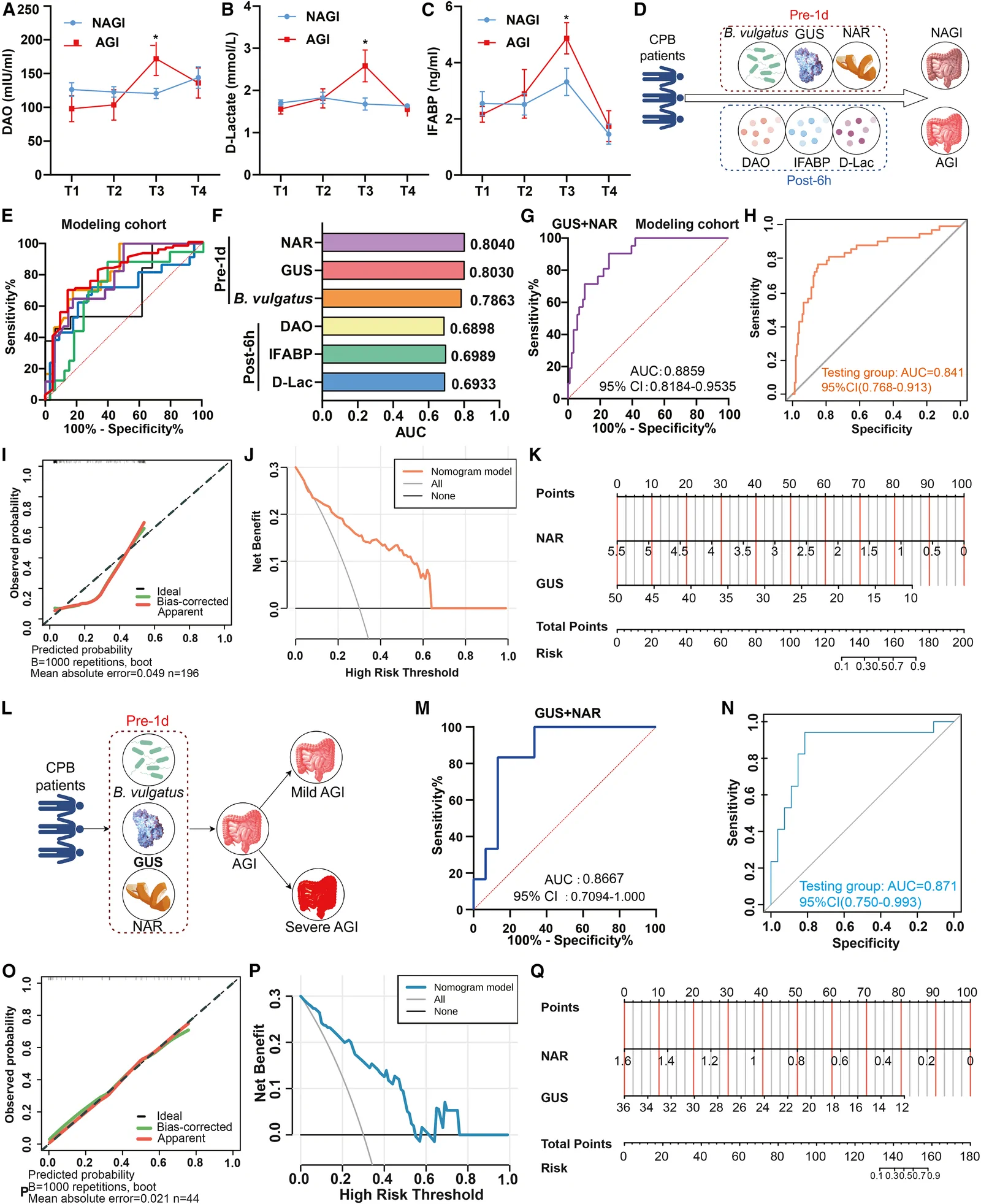
Preoperative fecal GUS activity and NAR predict postoperative AGI risk after CPB (A–C) Perioperative plasma DAO, D-lactate, and I-FABP levels in AGI (*n* = 21) and non-AGI (NAGI, *n* = 86) patients at T1 (preoperative), T2 (at the end of operation), T3 (6 h postoperatively), and T4 (24 h postoperatively). (D) Schematic overview for predictive modeling using preoperative fecal biomarkers. (E and F) ROC analyses of individual fecal biomarkers for AGI prediction. (G) ROC analysis evaluating the combined fecal biomarker model for AGI risk. (H) ROC curve in the independent internal validation cohort (*n* = 196; AGI, *n* = 44; NAGI, *n* = 152) for the NAR + GUS nomogram model. (I) Calibration plot validating the AGI prediction nomogram. (J) Decision curve analysis of the nomogram across threshold probabilities. (K) Nomogram tool for preoperative bedside AGI risk assessment. (L) Framework for distinguishing mild from severe AGI using preoperative biomarkers. (M and N) ROC validation for severe AGI prediction in both training and independent internal validation cohorts. (O) Calibration curve for validation of severe AGI severity prediction model. (P) Decision curve analysis for clinical decision-making at severe AGI thresholds. (Q) Nomogram for rapid bedside assessment of postoperative AGI severity. Quantitative measurements of plasma biomarkers (DAO, D-lactate, and I-FABP) were performed in triplicate. Quantitative data are presented as mean ± SEM where applicable. Statistical significance was defined as *p* < 0.05. AUCs with 95% CIs were estimated by nonparametric bootstrapping (B = 2,000) and compared by DeLong’s test. Logistic regression models were constructed using stepwise selection. Calibration was assessed by 1,000 bootstrap resamples and the Hosmer-Lemeshow test. Clinical utility was evaluated by decision-curve analysis. Abbreviations: DAO, diamine oxidase; D-LA, D-lactate; I-FABP, intestinal fatty acid binding protein; ROC, receiver operating characteristic; AUC, area under the curve; CI, confidence interval; SEM, standard error of the mean.

We therefore evaluated preoperative fecal microbial and metabolite markers for non-invasive risk prediction. Predictive models incorporating preoperative *B. vulgatus* abundance, GUS activity, and NAR content each demonstrated moderate-to-good predictive performance for postoperative AGI (AUCs: 0.786–0.804; Figures 7D–7F). Notably, a combined model integrating fecal NAR and GUS further improved predictive accuracy (AUC = 0.886) and showed higher predictive performance than individual plasma biomarkers (Figure 7G). In an independent internal validation cohort (*n* = 196), a nomogram integrating preoperative fecal NAR and GUS retained good discriminative performance (AUC = 0.841; sensitivity 77.3%, specificity 84.9%; Figure 7H), with good calibration (bootstrap mean absolute error [MAE] = 0.049, Hosmer-Lemeshow *p* > 0.05; Figure 7I). Decision curve analysis suggested potential net benefit across a wide range of risk thresholds, peaking at 40% (Figure 7J). The nomogram was further translated into a perioperative risk-scoring tool (Figure 7K).

To determine their value in severity stratification, we assessed the ability of these fecal biomarkers to distinguish between mild (AGI I–II) and severe (AGI III–IV) outcomes (Figure 7L). The nomogram exhibited high discriminative performance for severe AGI in both training (AUC = 0.867) and validation (AUC = 0.871) cohorts, with 100% sensitivity and 59.3% specificity in the independent internal validation cohort (Figures 7M and 7N). Calibration and decision curve analysis supported predictive reliability and potential clinical utility (bootstrap MAE = 0.021; Hosmer-Lemeshow *p* = 0.8449; Figures 7O and 7P). This risk stratification tool may facilitate early identification of patients at high risk for severe AGI in the perioperative setting (Figure 7Q).

Collectively, these data suggest that preoperative fecal GUS activity and NAR, which reflect microbe-host metabolic interactions, may serve as non-invasive biomarkers for preoperative prediction and severity stratification of AGI after CPB. This approach may help define a perioperative window for future microbiota-targeted intervention studies.

## Discussion

AGI remains a clinically important and incompletely resolved complication in patients undergoing CPB, with adverse consequences for postoperative recovery and long-term outcomes.^16^ Current efforts at early risk stratification have relied primarily on circulating biomarkers, such as I-FABP, DAO, citrulline, and D-lactate. However, these markers generally reflect epithelial injury after it has occurred and may be influenced by the complex physiological milieu following major surgery. This highlights the need for complementary preoperative tools that capture baseline host susceptibility and support earlier risk stratification. A better understanding of the potential contributors to AGI susceptibility may also inform exploratory monitoring approaches and future intervention studies.

Recent studies have positioned the gut microbiota as a crucial factor influencing outcomes after major surgery,^2^^,^^17^^,^^18^^,^^19^ yet the microbial functions and host pathways linking gut microbial communities to AGI remain incompletely defined. In this study, integrated metagenomic and metabolomic analyses, together with translational animal experiments, identified a microbe-metabolite-host axis associated with susceptibility to CPB-associated AGI. Evidence from GF mouse experiments showed that FMT from donors who did not develop postoperative AGI, as well as selective colonization with *B. vulgatus*, attenuated intestinal I/R injury. These findings support a functional contribution of specific microbial communities and metabolic activities to intestinal I/R injury resilience in this mouse model. However, the protective effect of such FMT is unlikely to be attributable solely to a single organism. While supplementation with *B. vulgatus* significantly reduced injury in AGI-FMT mice, *B. vulgatus* is more likely to act as an important component within a cooperative microbial ecosystem in which multiple taxa and metabolites collectively shape intestinal responses to ischemic stress. Moreover, although the microbial and metabolic signatures analyzed in this study were measured before surgery and before the clinical diagnosis of postoperative AGI, this temporal relationship alone does not establish that preoperative microbiota differences are independent causal determinants of AGI. These signatures may instead reflect baseline host susceptibility, unmeasured subclinical physiological states, medication exposure, diet, or other perioperative risk factors. Therefore, the human microbiome findings should be interpreted as identifying AGI-associated and predictive features, as well as candidate contributors to postoperative susceptibility, rather than proving independent causality. Longitudinal perioperative sampling and prospective validation studies will be needed to distinguish causal microbial contributors from biomarkers of susceptibility.

*B. vulgatus* has previously been associated with complex carbohydrate metabolism, mucin degradation, short-chain fatty acid production, and inflammatory or metabolic disorders.^20^^,^^21^^,^^22^^,^^23^ However, its direct role in acute intestinal epithelial injury had remained unclear. Our study extends this understanding by showing that both the abundance and enzymatic function of *B. vulgatus* are associated with protection against CPB-associated AGI. In particular, microbial GUS activity was associated with increased availability of free NAR, consistent with deconjugation of NAR-glucuronide. Prior studies have reported barrier-protective, anti-inflammatory, and antioxidative effects of NAR and related flavanones in models of systemic inflammation, hepatic injury, and cancer.^24^^,^^25^^,^^26^ By linking a defined microbial metabolic activity to epithelial protection, our findings emphasize the importance of functional microbial traits, rather than taxonomic abundance alone, in shaping host susceptibility to perioperative intestinal injury. Nevertheless, although these findings support a role for microbial GUS activity in increasing fecal free NAR availability, the biochemical pathway remains incompletely resolved. We did not perform isotope-resolved *in vivo* flux tracing to follow dietary NAR through host glucuronidation and subsequent microbial deconjugation, and therefore cannot fully exclude contributions from host intestinal or hepatic enzymes to NAR-glucuronide processing. In addition, although DSA reduced NAR-G-to-NAR conversion and attenuated *B. vulgatus*-associated protection, DSA-based inhibition should be interpreted as supportive rather than definitive evidence because pharmacological GUS inhibitors may have off-target effects. Future studies using stable isotope-labeled NAR/NAR-glucuronide and defined GUS-deficient or catalytically inactive bacterial mutants will be needed to directly establish the host-microbial flux of NAR metabolites *in vivo*.

Our data further suggest that *B. vulgatus*-associated GUS activity contributes to protection against intestinal I/R injury by increasing local free NAR availability. GUS is a key hydrolytic enzyme that catalyzes the release of bioactive aglycones from glucuronide conjugates, a process with well-established roles in drug metabolism and toxicity.^27^ Intestinal GUS activity is dynamic and context-dependent: while overall activity can be suppressed under inflammatory conditions,^28^ specific bacterial GUS enzymes can be upregulated and drive pathology in settings like drug-induced enteropathy.^29^ Furthermore, the gut microbial GUS repertoire is highly diverse in structure and function.^30^ In the setting of intestinal I/R injury, *B. vulgatus* appeared to deconjugate NAR-glucuronide and release free NAR within the intestinal lumen. Although *B. thetaiotaomicron* and GUS-overexpressing *E. coli* showed measurable GUS activity, fecal free NAR was more strongly enriched in the *B. vulgatus* condition. This suggests that *in vivo* NAR release is not determined solely by detectable GUS activity but may also depend on microbial abundance, substrate availability, enzyme accessibility, local metabolic context, and species-associated substrate preferences. These findings suggest that the protective effect may depend on species-associated context rather than GUS positivity alone, as *B. thetaiotaomicron*, despite being a phylogenetically related GUS-positive species,^31^ conferred limited protection in our models. Consistently, pharmacological inhibition of GUS with DSA largely abolished *B. vulgatu*s-mediated protection, whereas colonization with GUS-overexpressing *E. coli* partially recapitulated it. These findings indicate that GUS activity is an important component of the protective pathway, but that its efficacy depends on microbial species context and substrate-specific metabolic capacity. Nevertheless, because the current comparisons included only one additional GUS-producing *Bacteroides* species and engineered GUS-overexpressing *E. coli*, these data do not establish the uniqueness of *B. vulgatus* across the diverse gut GUS repertoire. Other GUS-producing commensals may also contribute to NAR-glucuronide deconjugation and intestinal protection. Future studies should systematically compare NAR-G-to-NAR conversion and protective capacity across diverse GUS-producing gut bacteria.

At the mechanistic level, our results implicate a TLR4/MyD88-SPRR2A-CHAC1 axis in NAR-associated epithelial protection. Our SPR data support direct binding of NAR to the TLR4/MD2 complex, and pharmacological disruption of TLR4/MyD88 signaling attenuated NAR-induced SPRR2A expression and its suppression of CHAC1-mediated glutathione depletion and ferroptosis. This mechanism provides a potential link between microbial flavanone metabolism and epithelial barrier preservation under ischemic stress. While the GPX4 or SLC7A11 pathways have been considered major nodal points in ferroptosis regulation,^32^^,^^33^ our data suggest that the SPRR2A-CHAC1 module may represent an additional regulatory layer in intestinal epithelial ferroptosis. Pharmacological inhibition of GUS or overexpression of *Chac1* reduced the protective effects of *B. vulgatus* or NAR, supporting the functional relevance of this pathway. Nevertheless, the structural determinants and context-dependent signaling outcomes of this interaction remain to be defined.

The clinical biomarker analyses further support the relevance of this microbe-metabolite axis to human AGI susceptibility. Preoperative fecal NAR and microbial GUS activity may reflect baseline intestinal resilience, whereas the early postoperative peak in serum I-FABP reflects acute epithelial injury after CPB. Patients with lower preoperative NAR likely sustained a more severe initial ischemic insult, as indicated by a higher 6-h I-FABP level, thereby increasing their risk of progressing to clinically overt AGI. This may help explain why NAR deficiency is associated with AGI risk despite the transient nature of circulating injury biomarkers. The observed variation in NAR availability may reflect host-microbiota metabolic interactions, although contributions from extrinsic factors cannot be fully excluded. Critically, comprehensive preoperative dietary assessment revealed no significant difference in flavonoid or general nutrient intake between patients who later developed AGI and those who did not. Although dietary assessment has inherent limitations, these data support the interpretation that reduced fecal NAR levels in AGI-prone individuals primarily reflect impaired microbial metabolic capacity, including reduced GUS activity, rather than differences in recent dietary exposure alone. We also evaluated preoperative PPI use, which showed a non-significant trend toward higher prevalence in patients who developed AGI. Because PPIs can alter gastrointestinal microbial ecology by reducing gastric acidity and reshaping downstream colonization patterns, differences in PPI exposure may have influenced baseline microbiota composition and related microbial functions. Although our cohort was not designed to determine the independent contribution of PPI use, this factor should be considered a potential confounder when interpreting the human microbiome findings. Future prospective studies should control for or stratify by PPI exposure.

Translating this mechanistic framework into clinical practice, we developed a preoperative risk stratification model based on fecal NAR concentration and GUS activity. This model showed better predictive performance than individual plasma biomarkers and retained good discrimination in an internal validation cohort. Importantly, this approach may enable identification of at-risk patients before clinically overt intestinal dysfunction develops, thereby supporting intensified monitoring or enrollment in future mechanism-guided interventional trials. However, these findings should not be construed as supporting immediate clinical implementation of *B. vulgatus*-based probiotics or NAR supplementation. Rather, they identify the NAR metabolic axis as a potentially modifiable pathway that warrants further clinical evaluation. Future studies are required to determine whether microbiome-based approaches, metabolite supplementation, dietary modulation, or other strategies targeting this pathway are safe, feasible, and effective in high-risk surgical populations.

In summary, our findings support a *B. vulgatus*-GUS-NAR metabolic axis that is associated with protection against CPB-associated AGI and links this axis to modulation of the SPRR2A-CHAC1 ferroptosis pathway. These findings provide mechanistic insight into how gut microbial metabolic function may shape intestinal susceptibility to perioperative ischemic injury and suggest that preoperative fecal GUS activity and NAR may serve as non-invasive biomarkers for AGI risk stratification. With further validation, microbiota-informed risk assessment may contribute to more individualized perioperative monitoring and may guide the design of future intervention studies in surgical and critically ill patients.

### Limitations of the study

Several limitations should be acknowledged. First, the clinical cohorts were derived from a single center with a modest sample size, and the fecal GUS/NAR-based prediction model has not yet been externally validated. Thus, its clinical utility remains preliminary, and larger multicenter prospective studies are needed to validate its generalizability and predictive performance. Dietary flavanone exposure was assessed retrospectively by questionnaire, which may not fully account for individual dietary variability or unmeasured confounders. Although both male and female patients were enrolled, the study was not powered for sex-stratified analyses; potential sex-related influences on the identified biomarkers therefore cannot be fully excluded. Moreover, although our experimental models support a link between NAR availability and epithelial ferroptosis regulation, we did not directly establish a mechanistic association between preoperative fecal NAR levels and ferroptosis-related epithelial changes in human intestinal samples. Second, the lack of continuous, high-frequency biomarker monitoring limited our ability to define the temporal relationships among circulating injury markers, microbial metabolic activity, fecal NAR availability, and clinical AGI onset. Future studies integrating serial biomarker measurements with imaging or spatial omics approaches may clarify the transition from early epithelial injury to established intestinal dysfunction. Third, the contribution of *B. vulgatus* to NAR-glucuronide deconjugation should be interpreted cautiously rather than as evidence of strict bacterial specificity. Because the current study did not systematically compare NAR-G-to-NAR conversion across a broader panel of GUS-positive gut bacteria, including controls such as *B. thetaiotaomicron* or engineered GUS-overexpressing *E. coli*, we cannot exclude the possibility that other commensals with GUS activity may also mediate this reaction. Thus, our data support a species-associated contribution of *B. vulgatus* under the tested experimental conditions, rather than a uniquely exclusive role. Future studies will be required to define the relative contribution of diverse GUS-producing gut microbes to free NAR release *in vivo*. In addition, only male mice were used to minimize estrous cycle-related variability and to align with prior intestinal I/R studies; validation in female mice remains an important future direction. Finally, although the protective effects of *B. vulgatus* and NAR were demonstrated in preclinical models, their safety and efficacy in humans require prospective validation.

## Resource availability

### Lead contact

Requests for further information and resources and reagents should be directed to and will be fulfilled by the lead contact, Kexuan Liu (liukexuan705@163.com).

### Materials availability

Mouse lines and all unique reagents used in this study are available from the lead contact with a completed Material Transfer Agreement.

### Data and code availability

• The raw 16S rRNA sequencing data have been deposited in the NCBI Sequence Read Archive (SRA) under accession numbers PRJNA1307018, PRJNA1248219, and PRJNA1248385. Transcriptomic data are available in the SRA under accession number PRJNA1298395. The metabolomics data generated in this study have been deposited in the Metabolomics Workbench repository under Study ID: ST005181. These data are publicly available at https://doi.org/10.21228/M8TV9C.
• This paper does not report original code.
• Any additional information required to reanalyze the data reported in this work paper is available from the lead contact upon request.

## Acknowledgments

This study was supported by the 10.13039/501100001809National Natural Science Foundation of China (grant numbers 82372169, 82202361, and 82572476 to F.D.; 82330067 to K.L.; 82472185 to J.H.; 82504493 to H.L.), the China International Medical Exchange Foundation (grant number Z-2017-24-2421 to F.D.), the Guangzhou Basic and Applied Basic Research Project Special Fund (Young Doctoral “Qihang” Program; grant number SL2024A04J01443 to F.D.), the Southern Medical University Distinguished Recruit Talent Research Startup Fund (grant number R10101063 to F.D.), and the Nanfang Hospital President’s Fund (grant number K50112078 to F.D.). The authors acknowledge the Metabolomics Workbench for facilitating data deposition and access and the NIH Common Fund National Metabolomics Data Repository (NMDR) for data curation. The Metabolomics Workbench is supported by NIH grants U2C DK119886 and OT2 OD030544.

## Author contributions

F.D., J.H., Z. Li, H.Z., and K.L. conceived and designed the study. J.H., Y.M., S.X., Z. Lin, Q.S., S.H., and W.T. developed the methodology. Experimental work was carried out by Y.M. (mouse I/R models), S.X. (molecular experiments), Z. Lin (clinical data analysis), and L.X. (metabolomics profiling). S.F., L.W., X.C., H.L., Z.Z., and J.H. performed formal analysis, including 16S rRNA gene sequencing, transcriptomic analysis, and statistical validation. J.H., F.D., and K.L. acquired funding. F.D. and J.H. drafted the manuscript. Y.M., S.X., Z.L., and K.L. provided critical revision of the manuscript. F.D. and K.L. supervised the study. All authors reviewed and approved the final manuscript.

## Declaration of interests

The authors declare no competing interests.

## Declaration of generative AI and AI-assisted technologies in the manuscript preparation process

During the preparation of this manuscript, the authors used ChatGPT by OpenAI solely for language editing, including grammar, clarity, and brevity. The authors reviewed and edited the content as needed and take full responsibility for the final content of the manuscript.

## STAR★Methods

### Key resources table

**Table:** 

REAGENT or RESOURCE	SOURCE	IDENTIFIER
**Antibodies**		

Rabbit polyclonal to ZO1	Abcam	Cat# ab216880; RRID: AB_2909434
Rabbit monoclonal to Occludin	Abcam	Cat# ab216327; RRID: AB_2737295
Rabbit monoclonal anti-Ki67	Abcam	Cat# ab16667; RRID: AB_302459
Goat anti-rabbit HRP	Abcam	Cat# ab6721; RRID: AB_955447
Rabbit anti-CHAC1	Abcam	Cat# ab217808; RRID: AB_3678643
Rabbit anti-SPRR2A	Thermo Fisher Scientific	Cat# PA5-107027; RRID: AB_2817743
Rabbit anti-GPX4	Thermo Fisher Scientific	Cat# MA5-32827; RRID: AB_2810103
Rabbit anti-COX-2	Thermo Fisher Scientific	Cat# MA5-14568; RRID: AB_10984436
Rabbit anti-TLR4	Thermo Fisher Scientific	Cat# 14-9924-82; RRID: AB_468617
Rabbit anti-MYD88	Thermo Fisher Scientific	Cat# PA5-19919; RRID: AB_11154287

**Bacterial and virus strains**		

*Bacteroides vulgatus*	ATCC	ATCC 8482
*Bacteroides thetaiotaomicron*	ATCC	ATCC 29148

**Biological samples**		

Human blood samples	Nanfang Hospital, Southern Medical University	N/A

**Chemicals, peptides, and recombinant proteins**		

Recombinant human TLR4 protein	MedChemExpress	Cat# HY-P73586
Recombinant human CHAC1 protein	MedChemExpress	Cat# HY-P701945
Recombinant human SPRR2A	IPODIX	Cat# PA2000-1222
Dihydroethidium	Thermo Fisher Scientific	Cat# D11347
Accutase	Stemcell	Cat# 07920
Liperfluo	Dojindo	Cat# L248-10
Vancomycin	Macklin	Cat# 1404-93-9
Neomycin	Macklin	Cat# 1405-10-3
Metronidazole	Macklin	Cat# 443-48-1
Ampicillin	Macklin	Cat# 69-53-4
TRIZOL	Invitrogen	Cat# 15596026
TJ-M2010-5	MedChemExpress	Cat# HY-139397
Naringenin	MedChemExpress	Cat# HY-N0100
D-saccharic acid-1,4-lactone	MedChemExpress	Cat# HY-134453A
Naringenin-7-*O*-β-D-glucuronide	Mlbio	Cat# J21597

**Critical commercial assays**		

Cell Counting Kit-8	Dojindo	Cat# CK04
Lactate dehydrogenase (LDH) assays	Nanjing Jiancheng	Cat# A020
Malondialdehyde (MDA) detection kit	Solarbio	Cat# BC0025
Lipid peroxide (LPO) detection kit	Solarbio	Cat# BC5245
T-GSH/GSSG Assay Kit	Nanjing Jiancheng	Cat# A061-1-2
Fe^2+^ Assay Kit	Solarbio	Cat# BC4355
Cell ferrous iron colorimetric Assay Kit	Elabscience	Cat# E-BC-K881-M
β-glucuronidase Activity Assay Kit	Solarbio	Cat# BC2560
Intestinal fatty acid-binding protein ELISA Kit	Cusabio	Cat# CSB-E08024h
Citrulline ELISA Kit	USCN	Cat# UCEA505Ge
Diamine oxidase ELISA Kit	Cusabio	Cat# CSB-E10137h

**Deposited data**		

Raw sequencing data	https://www.ncbi.nim.nih.gov/sra	SRA: PRJNA1307018, PRJNA1248219, PRJNA1248385
Transcriptomic data	https://www.ncbi.nlm.nih.gov/sra	SRA: PRJNA1298395
Metabolomics data	https://www.metabolomicsworkbench.org	Metabolomics Workbench: ST005181; https://doi.org/10.21228/M8TV9C

**Experimental models: Cell lines**		

Intestinal organoids	This paper	N/A

**Experimental models: Organisms/strains**		

Germ-free mice	Cyagen Biosciences Inc	N/A
*Sprr2a* knockout (*Sprr2a*-KO) mice	Cyagen Biosciences Inc	N/A
Male C57BL/6J mice	Animal Center of Nanfang Hospital, Southern Medical University	N/A
*Chac1*-overexpressing (*Chac1*-OE) mice	Cyagen Biosciences Inc	N/A
*Chac1* knockout (*Chac1*-KO) mice	Cyagen Biosciences Inc	N/A

**Oligonucleotides**		

Primers for RT–PCR, see Table S3	This paper	N/A

**Software and algorithms**		

ImageJ	National Institutes of Health	https://imagej.nih.gov/ij/index.html
Origin 9.0	OriginLab	OriginLab
TraceFinderTM	Thermo Fisher Scientific Corp	https://www.thermofisher.cn/order/catalog/product/OPTON-31001
GraphPad Prism 9	GraphPad	https://www.graphpad.com/
Picrust2	Picrust2 Software	https://picrust.github.io/picrust/
FlowJo, v10.8	BD Biosciences	https://www.flowjo.com/
PlexArray HT software	PlexArray™	https://plexera.com/

### Experimental model and study participant details

#### Ethics and clinical research protocol

This study was conducted in accordance with the ethical standards set forth in the Declaration of Helsinki and received approval from the Institutional Review Board of Nanfang Hospital, Southern Medical University (Approval No.: NFEC-202009-k2-01). Consecutive patients undergoing elective heart valve replacement or coronary artery bypass grafting under cardiopulmonary bypass were prospectively enrolled between July 2019 and February 2025 in the Department of Cardiac Surgery, Nanfang Hospital, Southern Medical University, Guangzhou, China. Written informed consent was obtained from all participants prior to enrollment.

#### AGI assessment and cohort stratification enrollment criteria

Participants were excluded if they met any of the following criteria: (1) age younger than 18 years or older than 75 years; (2) severe hepatic or renal dysfunction, defined as ALT/AST greater than three times the upper limit of normal or estimated glomerular filtration rate (eGFR) less than 60 mL/min/1.73m^2^; (3) a history of chronic digestive diseases, prior gastrointestinal surgery, or confirmed/suspected intestinal ischemia or necrosis; and (4) use of antidiarrheals, laxatives, or prebiotics within one week prior to enrollment, or antibiotics within the previous three months.

For the discovery (derivation) cohort, the study employed *a priori* power analysis based on AGI incidence rates documented in prior studies (≈20%). To achieve 90% power at α = 0.05 to detect significant biomarker differences, a minimum of 104 patients was required. Ultimately, 107 patients were enrolled after accounting for potential exclusions. Of these, 45 patients (42.06%) were male and 62 (57.94%) were female. In the AGI group, 5/21 (23.81%) were male and 16/21 (76.19%) were female; in the NAGI group, 40/86 (46.51%) were male and 46/86 (53.49%) were female. Patient sex was recorded from medical records, and gender was not separately collected. To ensure robustness, an independent internal validation cohort of 196 patients was recruited. This aligned with biomarker validation standards requiring ≥150 cases to achieve >80% power for AUC >0.75 (per TRIPOD guidelines). This cohort confirmed the predictive accuracy of fecal naringenin +β-glucuronidase (GUS) activity.

AGI diagnosis and severity were strictly based on the aforementioned ESICM consensus,^34^ guided by daily evaluations of clinical symptoms (e.g., abdominal distension, vomiting), enteral nutrition tolerance, and abdominal computed tomography (CT) findings within 72 h post-CPB. According to the ESICM consensus, AGI is classified into four grades based on increasing severity: Grade I (at risk) refers to transient, self-limiting gastrointestinal dysfunction (such as postoperative nausea and vomiting, or absence of bowel sounds); Grade II (gastrointestinal dysfunction) indicates dysfunction requiring specific interventions (such as feeding intolerance or moderate intra-abdominal hypertension); Grade III (gastrointestinal failure) refers to failure that does not respond to interventions, persists, and leads to the exacerbation of multiple organ dysfunction syndrome (MODS); Grade IV (life-threatening gastrointestinal failure) denotes severe, fatal failure accompanied by direct remote organ involvement (such as intestinal ischemia and necrosis, massive hemorrhage, or abdominal compartment syndrome).

In this study, patients meeting the criteria for AGI grade I or higher according to the ESICM standards were classified into the AGI group, while those without signs of gastrointestinal dysfunction were classified into the non-AGI (NAGI) group. In the derivation cohort, 21 patients were classified as AGI and 86 as NAGI; in the independent internal validation cohort, 44 patients were assigned to the AGI group and 152 to the NAGI group.

Peripheral blood samples were collected at four time points: preoperatively (T1), at the conclusion of surgery (T2), 6 h postoperatively (T3), and 24 h postoperatively (T4). These samples were analyzed for intestinal fatty acid-binding protein (IFABP; Cusabio, Wuhan, China), citrulline (USCN, Wuhan, China), and diamine oxidase (DAO; Cusabio, Wuhan, China). Preoperative fecal samples (collected one day before surgery) were also obtained to measure NAR levels, GUS activity, and the abundance of *Bacteroides vulgatus* (*B. vulgatus*).

#### Quality control and methodological rigor

A rigorous three-tiered quality assurance system was implemented, comprising multi-level biomarker validation (serum assay CV <5%), dual-blinded AGI assessment by intensivists (Cohen’s κ > 0.8), and real-time perioperative medication monitoring via electronic health records. Missing data were addressed through standardized follow-up protocols. Baseline characteristics between completers and non-completers were compared to evaluate selection bias, with primary analyses following an available-case approach. Biospecimens were processed and frozen at −80°C within 15 min post-collection to ensure metabolite stability. The protocol adhered to international (ESICM) guidelines and landmark studies, with comprehensive informed consent procedures addressing research objectives, biospecimen handling, and data confidentiality through anonymized coding and restricted usage. Oversight by an independent Data Safety Monitoring Committee ensured encrypted data storage, traceable sample management, and mitigation of therapeutic misconception, with no additional medical or financial burden imposed on participants.

#### Patient and public involvement

Patients or the public were not involved in the design, or conduct, or reporting, or dissemination plans of our research.

#### Experimental animals

All animal experiments were approved by the Institutional Animal Care and Use Committee (IACUC) of Nanfang Hospital, Southern Medical University (IACUC-LAC-20221117-010), and conducted in strict accordance with the ARRIVE Guidelines and the General Principles of Laboratory Animal Welfare and Ethics (GB/T 35892-2018). Male C57BL/6J wild-type (WT) mice (8–10 weeks old, 22–25 g) were procured from the Animal Center of Nanfang Hospital, Southern Medical University (Guangzhou, China). Male germ-free (GF) mice, intestinal epithelium–specific *Sprr2a* knockout (*Sprr2a*-KO) mice, as well as *Chac1*-overexpressing (*Chac1*-OE) and *Chac1* knockout (*Chac1*-KO) mice (8–10 weeks old, 22–25 g), were obtained from Cyagen Biosciences (Suzhou, China). All animals were housed in specific pathogen-free (SPF) barrier facilities under standardized environmental conditions (22 ± 1°C, 50 ± 10% humidity, 12-h light/dark cycle) with unrestricted access to autoclaved food and water.

#### Intestinal I/R modeling

A well-established murine model of intestinal ischemia-reperfusion (I/R) injury was utilized in this study.^35^ Mice were anesthetized with 3% isoflurane for induction and maintained with 1.5% isoflurane throughout the procedure. Core body temperature was maintained at 37.0°C using a heating pad. Under sterile conditions, a midline laparotomy was performed to expose and isolate the superior mesenteric artery (SMA), which was subsequently occluded with a non-traumatic microvascular clip (RWD Life Science, USA). Simultaneously, 500 μL of normal saline was administered subcutaneously for fluid resuscitation. Following 60 min of ischemia, the vascular clip was removed to initiate reperfusion, and an additional 500 μL of normal saline was injected subcutaneously. Vital signs were continuously monitored during reperfusion. Two hours after reperfusion, whole blood was collected via cardiac puncture under anesthesia and tissue samples from the terminal ileum, cecum, liver, lungs, and kidneys were harvested. All specimens were immediately processed for subsequent analyses.

All mice that successfully underwent the intestinal I/R modeling procedure (confirmed by immediate intestinal blanching upon SMA occlusion and blood flow restoration after clip release) were included in the study. Animals were excluded only if death occurred prior to the predetermined sampling endpoint (2 h post-reperfusion). No data points were excluded. All criteria were established prior to the experiment.

#### Small intestinal organoid isolation, culture, and *in vitro* hypoxia-reoxygenation (H/R) model establishment

The isolation and culture of small intestinal organoids were performed as previously described.^14^ Briefly, following euthanasia of WT and *Chac1*-OE mice, a 20-cm segment of proximal small intestine was harvested, rinsed in cold PBS to remove mesentery and adipose tissue, longitudinally opened, cleaned, and sectioned into 2 mm fragments. Tissue fragments were vigorously pipetted until the supernatant became clear, then digested with a gentle cell dissociation reagent (25 mL; 20 rpm; 15 min; room temperature). The resultant suspension was filtered through a 70 μm strainer after resuspension in PBS with 0.1% BSA. Isolated crypts were embedded in Matrigel (STEMCELL Technologies Inc.), solidified at 37°C for 40 min, and maintained in IntestiCult medium (STEMCELL Technologies Inc.) under standard conditions (37°C, 5% CO_2_, 20% O_2_). Organoid identity was confirmed by qPCR detection of the intestinal stem cell marker *Lgr5*, and structural integrity was assessed by H&E staining. All cultures tested negative for mycoplasma contamination using a MycAway Plus-Color One-Step Mycoplasma Detection Kit (Yeasen, Shanghai, China).

On day 5 of culture, organoids were subjected to the hypoxia-reoxygenation (H/R) challenge: hypoxia was induced by incubation at 1% O_2_, 5% CO_2_, and 37°C for 12 h in a tri-gas incubator, followed by reoxygenation under normoxic conditions (20% O_2_, 5% CO_2_, 37°C) for 4 h. Prior to H/R induction, organoids derived from both WT and *Chac1*-OE mice were pretreated for 2 h with either 10% *B. vulgatus* culture supernatant or 80 μM naringenin (NAR).^36^^,^^37^ After the H/R procedure, both organoids and their culture supernatants were collected for subsequent assessment of injury and viability.

#### Culture conditions for *B. vulgatus*

*B. vulgatus* ATCC 8482 (BeNa Culture Collection, Beijing, China) lyophilized powder was reconstituted in 0.5 mL sterile PBS and plated on blood agar. After 48 h of anaerobic incubation at 37°C, a single colony was selected and inoculated into 5 mL fluid thioglycollate medium (FTM) for an additional 48 h under anaerobic conditions. When the culture reached a density of 1 × 10^9^ CFU/mL, as determined by plate counting, the bacterial suspension was mixed with an equal volume of 25% (v/v) glycerol and stored at −80°C for subsequent use. To prepare the working bacterial suspension, 100 μL of frozen stock was inoculated into 5 mL fresh FTM and cultured anaerobically at 37°C for 48 h. For *in vivo* experiments, 200 μL of live *B. vulgatus* (1 × 10^9^ CFU/mL) was administered to mice by oral gavage. For preparation of heat-killed *B. vulgatus*, the live bacterial suspension was incubated at 70°C for 30 min.

#### Culture conditions for *B. thetaiotaomicron*

*Bacteroides thetaiotaomicron* (ATCC 29148) was grown anaerobically in trypticase-yeast extract-glucose (TYG) medium at 37°C. Cultures were initiated by diluting frozen stocks 1% into pre-reduced medium and incubating until approximately 1 × 10^9^ CFU/mL. For *in vivo* use, mice received 200 μL of bacterial suspension. Long-term storage utilized 25% glycerol stocks at −80°C.

#### Construction and culture of engineered *E. coli*^*GUS*^

To generate a gain-of-function control strain with enhanced GUS activity while minimizing background from endogenous *E. coli* β-glucuronidase, an engineered *E. coli* strain was constructed. The native uidA locus in the parental strain *E. coli* MG1655 was replaced with a gusA expression cassette derived from *B. vulgatus* ATCC 8482, resulting in the final strain *E. coli*^*GUS*^. This strain was commercially generated and verified by Forhighbio (Hangzhou, China). Correct genomic integration of the gusA cassette was confirmed by the manufacturer via PCR across the insertion junctions and subsequent Sanger sequencing.

The engineered *E. coli* strain was cultured aerobically in Luria-Bertani (LB) medium at 37°C to a mid-log phase OD_600_ of 0.8–1.0, corresponding to a concentration of approximately 1 × 10^9^ CFU/mL. For experiments, mice were administered 200 μL of bacterial suspension; cryopreservation was achieved in 25% glycerol at −80°C.

### Method details

#### Animal experimental design

##### Gut microbiota and GUS activity on NAR metabolism

To investigate the impact of gut microbiota and GUS activity on NAR metabolism, male wild-type (WT) mice (*n* = 24) were randomly assigned to three groups (*n* = 8 per group)(Based on power analysis and similar studies in the field): Control (receiving PBS by oral gavage daily for 1 week), Antibiotics (receiving vancomycin [100 mg/kg], neomycin [200 mg/kg], metronidazole [200 mg/kg], and ampicillin [200 mg/kg] by oral gavage daily for 1 week to deplete the gut microbiota),^38^ and DSA (receiving D-saccharic acid-1,4-lactone [DSA, MedChemExpress, 20 mg/kg/day, p.o.] as a GUS inhibitor for 1 week). Following treatment, fecal samples were collected for the assessment of GUS activity and NAR levels prior to intestinal I/R induction.

##### Role of GUS and NAR in intestinal I/R injury

To determine the effects of GUS and NAR on intestinal I/R injury, male WT mice (*n* = 32) were randomly divided into four groups (*n* = 8 per group): Sham (laparotomy without vascular occlusion), I/R, I/R + NAR (receiving NAR [50 mg/kg, MedChemExpress] intraperitoneally 1 h before I/R), and I/R + DSA (receiving DSA [20 mg/kg, p.o.] once daily for 7 days before I/R).^24^^,^^39^ Postoperative collection of blood, ileum, and cecum samples was performed under aseptic conditions for further analyses.

##### Fecal microbiota transplantation

Fecal microbiota transplantation (FMT) was performed as previously described to assess whether preoperative microbiota could functionally modulate susceptibility to intestinal I/R injury in recipient mice.^40^ Fresh preoperative fecal samples were collected from patients in the NAGI and AGI groups undergoing CPB and pooled separately by group. The feces were resuspended in PBS (0.125 g/mL), and 100 μL of the suspension was gavaged daily into GF recipient mice for 7 days. Mice receiving fecal transplants from these preoperative donors were designated NAGI-FMT and AGI-FMT groups, respectively. One week after FMT, an additional group of AGI-FMT recipients was supplemented with *B. vulgatus* (1 × 10^9^ CFU/mL) daily for 3 consecutive days prior to I/R challenge.

##### Role of *B. vulgatus* and its relationship to NAR in I/R injury

The role of *B. vulgatus* in I/R injury and its interaction with NAR was evaluated using both WT and GF mice. WT mice (*n* = 32) were randomly allocated to four groups: Sham, I/R, I/R + *B. vulgatus* (receiving 200 μL of live *B. vulgatus* suspension [1 × 10^9^ CFU/mL] by oral gavage daily for 1 week), and I/R + K. *B. vulgatus* (same dose of heat-killed *B. vulgatus*, inactivated at 70°C for 30 min). GF mice (*n* = 16) were divided into I/R and I/R + *B. vulgatus* groups (same dose, gavaged for 3 days). Fecal samples from GF mice under control, *B. vulgatus*-treated, and DSA-treated (20 mg/kg/day, p.o., for 3 days) conditions were collected for NAR quantification. Additionally, NAR levels were measured in culture supernatants, including FTM medium, 48 h anaerobic cultures of *B. vulgatus*, and 48 h cultures supplemented with 1.5 mg/mL DSA.^41^

##### Experimental design for validation of species-associated GUS activity and GUS dependency

To assess the role of *B. vulgatus* and its GUS-dependent mechanism in intestinal I/R injury, mono-colonization studies were performed in GF mice. Male GF mice were maintained under sterile isolator conditions and randomly assigned to six groups (*n* = 8 per group): (1) GF + I/R (sterile control); (2) GF + *B*. *vulgatus* + I/R; (3) GF + *B*.*thetaiotaomicron* + I/R (GUS-positive comparator); (4) GF + *E. coli*^GUS^ + I/R (GUS gain-of-function control); (5) GF + *B*. *vulgatus*+ DSA+I/R (DSA 20 mg/kg/day, p.o., 3 days; GUS loss-of-function control); and (6) WT + I/R (physiological reference). All GF groups received a single oral gavage of live bacteria (200 μL, 1 × 10^9^ CFU/mL) and were maintained for 3 days to establish colonization before I/R surgery. After I/R, fecal samples, ileal tissues, and cecal contents were collected for assessment of GUS activity and NAR levels, histopathological scoring, and barrier function evaluation, respectively.

##### *In vitro* assessment of bacterial GUS activity and NAR generation

To evaluate species-associated GUS activity and its capacity to generate NAR, *in vitro* mono-culture assays were performed. The following groups were included (*n* = 4 per group): (1) *B. vulgatus* cultured in FTM; (2) *B. thetaiotaomicron* cultured in TYG medium; (3) Engineered *E. coli*^*GUS*^ (GUS gain-of-function control) cultured in LB medium; (4) *B. vulgatus* in FTM + DSA (1.5 mg/mL); (5) Corresponding sterile culture media as negative controls (FTM, TYG, and LB medium). All bacterial strains were cultured under appropriate conditions (*Bacteroides* anaerobically, *E. coli* aerobically) at 37°C for 48 h. At the end of incubation, bacterial suspensions were collected for GUS activity assay, and culture supernatants were harvested for NAR quantification by LC-MS/MS.

##### *In vivo* validation of GUS-dependent NAR-G deglucuronidation by *B. vulgatus*

To test whether *B. vulgatus* contributes to GUS-mediated naringenin-7-*O*-β-D-glucuronide (NAR-G) deglucuronidation *in vivo*, GF mice were randomly assigned to three groups (*n* = 4 per group per time point): (1) GF; (2) GF + *B. vulgatus*; (3) GF + *B. vulgatus* + DSA (20 mg/kg/day, p.o., for 3 days). On day 3 post-colonization, mice received an oral bolus of NAR-G (10 mg/kg). Fecal samples were collected at 0, 1, 2, 4, 8, and 12 h post-administration for measurement of GUS activity, free NAR, residual NAR-G, and calculation of the free NAR/NAR-G ratio. Pharmacokinetic parameters, including Peak fecal concentration and area under the curve (AUC), were derived from the time course data.

##### *In vitro* validation of GUS-dependent NAR-G deglucuronidation by *B. vulgatus*

To test whether *B. vulgatus* contributes to free NAR generation through GUS-mediated deglucuronidation of NAR-G *in vitro*, a time course assay was performed with the following groups (*n* = 4 per group per time point): (1) *B. vulgatus* alone; (2) *B. vulgatus* + NAR-G; (3) *B. vulgatus* + NAR-G + DSA; (4) Heat-killed *B. vulgatus* + NAR-G; (5) FTM + NAR-G; (6) FTM + NAR standard (500 ng/mL); (7) FTM. *B. vulgatus* was cultured anaerobically in FTM medium at 37°C for 48 h and adjusted to 1 × 10^9^ CFU/mL. Heat-killed controls were prepared by incubating bacterial suspension at 70°C for 30 min. NAR-G (100 μM) and DSA (1.5 mg/mL) were added as designated. All groups were incubated anaerobically at 37°C for 24 h, with samples collected at 0, 6, 12, and 24 h for GUS activity assay and LC-MS/MS quantification of free NAR and residual NAR-G in culture supernatants. NAR-G consumption was assessed by substrate depletion over time.

##### TLR4/MyD88 pathway investigation

To assess the involvement of the TLR4/MyD88 pathway in the protective effects of *B. vulgatus* and NAR, WT mice were pretreated for 1 week with either vehicle (1% DMSO, i.p.) or the MyD88 inhibitor TJ-M2010-5 (50 mg/kg, i.p.).^42^ Each pretreatment group was subdivided into three experimental subgroups: I/R, I/R + *B. vulgatus*, and I/R + NAR, with all interventions administered during the 7-day pretreatment period.

##### Role of *Sprr2a* and *Chac1* in *B. vulgatus*/NAR protection against I/R injury

To elucidate the roles of *Sprr2a* and *Chac1* in *B. vulgatus*/NAR-mediated protection against intestinal I/R injury, WT, *Sprr2a* knockout (*Sprr2a*-KO), and *Chac1* overexpression (*Chac1*-OE) mice were assigned to I/R, I/R + *B. vulgatus*, or I/R + NAR groups (*n* = 8 per group) for groupwise comparisons.

##### 16S rRNA gene sequencing and data analysis

Fecal microbial DNA was extracted using the HiPure Stool DNA Kit (Magen, China). The V3–V4 region of the bacterial 16S rRNA gene was amplified using primers 341F (5′-CCTACGGGNGGCWGCAG-3′) and 806R (5′-GGACTACHVGGGTATCTAAT-3′). PCR products were used to construct sequencing libraries with the Illumina DNA Prep Kit, assessed for quality on an ABI StepOnePlus system, and subjected to high-throughput sequencing on an Illumina NovaSeq 6000 platform with paired-end sequencing (LC-Bio Technology Co., Ltd, China). Following quality control, valid sequences were clustered into operational taxonomic units (OTUs) at a 97% similarity threshold using UPARSE implemented in USEARCH v9.2.64, with the most abundant sequence representing each OTU.^43^ Taxonomic classification was performed with the RDP classifier referencing the SILVA database (v138.2) at a confidence threshold of 0.8–1. Alpha diversity metrics (Chao1, ACE, Shannon, Simpson, Good’s coverage, and Pielou’s evenness) were calculated in R (v4.0.3). Beta diversity was evaluated by principal coordinate analysis (PCoA) based on Bray-Curtis dissimilarity matrices using the vegan package (v2.4.3). Differential abundance among groups was analyzed using Welch’s *t* test, Wilcoxon test, Tukey’s HSD test, and Kruskal-Wallis test. Biomarker species were identified via linear discriminant analysis effect size (LEfSe) and random forest analysis, and functional predictions for KEGG pathways were inferred using PICRUSt2.

##### Non-targeted metabolomics assays and data analysis

Fecal metabolites were extracted from 100 mg of sample using ice-cold methanol/acetonitrile/water (2:2:1, v/v/v), followed by ultrasonication and centrifugation. The resulting supernatant was filtered, concentrated, and analyzed with a Vanquish UHPLC system coupled to an Orbitrap Exploris 480 high-resolution mass spectrometer (Thermo Fisher Scientific). Chromatographic separation was carried out on an ACQUITY UPLC BEH Amide column (2.1 mm × 100 mm, 1.7 μm; Waters) under HILIC conditions, with mobile phases A (25 mM ammonium acetate and 25 mM ammonium hydroxide in water) and B (acetonitrile) at a flow rate of 0.4 mL/min. Mass spectrometric detection utilized electrospray ionization (ESI) in both positive and negative ion modes across a mass range of m/z 70–1200. Raw data were processed with Progenesis QI for peak detection, retention time alignment, and normalization; features present in less than 50% of samples were excluded. After internal standard correction, metabolites were identified by comparison to HMDB 4.0 using a mass tolerance of 5 ppm and MS/MS score ≥0.7. Multivariate statistical analysis (OPLS-DA, VIP ≥1.0) was performed with SIMCA-P, and differential metabolites were identified by combining t-tests (*p* < 0.05, FDR<0.05). Metabolic pathway enrichment analysis was conducted using MetaboAnalyst 5.0 (FDR<0.05).

##### RNA sequencing and data analysis

Total RNA was isolated from intestinal tissues of the I/R, I/R + *B. vulgatus*, and I/R + NAR groups using TRIZOL reagent (Invitrogen, Carlsbad, CA, USA), and RNA integrity was verified by denaturing agarose gel electrophoresis. RNA was fragmented and reverse-transcribed into cDNA with SuperScript II Reverse Transcriptase (Invitrogen, cat. 1896649, USA). The resulting cDNA was used to construct sequencing libraries with an average insert size of 300 ± 50 bp. Sequencing was performed on the Illumina NovaSeq 6000 platform (LC-Bio, China), generating paired-end reads (2 × 150 bp) according to the manufacturer’s protocol. Raw sequencing reads were quality-filtered using fastp, aligned to the reference genome with HISAT2, and assembled with StringTie. Gene expression quantification was conducted using RSEM. Principal component analysis (PCA) and correlation analysis were employed to evaluate sample relationships. Differentially expressed mRNAs (DEMs) were identified using edgeR, with thresholds of |log_2_ fold change| > 1 and *p* < 0.05. Functional annotation of DEMs was performed using Gene Ontology (GO) and KEGG pathway enrichment analyses in the clusterProfiler R package to elucidate biological functions and pathways.

##### Quantitative analysis of NAR and NAR-G

NAR and NAR-G levels were quantified by LC-MS/MS as previously described.^44^ Briefly, 100 mg of fecal sample was homogenized in ice-cold water (1:9, w/v), sonicated for 15 min, and centrifuged at 12,000 rpm for 10 min at 4°C. Culture supernatants were collected directly. Supernatants were mixed with methanol (1:4, v/v), vortexed, centrifuged, and filtered (0.22 μm) prior to LC-MS/MS analysis. Chromatographic separation used a C18 column with mobile phase of 0.1% formic acid in water (A) and acetonitrile (B). MS detection was performed in multiple reaction monitoring mode with electrospray ionization in negative/positive ion mode. Calibration curves were generated using NAR standards (1, 20, 100, 500, and 1000 ng/mL) and NAR-G standards (10, 200, 1000, 5000, and 10,000 ng/mL) in methanol. Data acquisition and quantitative analysis were performed using TraceFinder software version 3.3 sp1 (Thermo Fisher Scientific, USA). Concentrations were normalized to sample weight or volume as appropriate.

##### β-glucuronidase activity assay

β-glucuronidase (GUS) activity was measured in bacterial pellets, fecal samples, and human samples using a commercial kit (BC2560, Solarbio, China) following the manufacturer’s instructions with minor modifications. Bacterial pellets were collected by centrifugation, washed twice with PBS, and resuspended in assay buffer. Freshly collected fecal samples were immediately frozen at −80°C until analysis. Fecal samples were homogenized in assay buffer and centrifuged to obtain supernatants. All measurements were performed in triplicate with appropriate standards and controls.

##### Availability and assessment of preoperative dietary data

To evaluate potential dietary confounding, preoperative intake of flavonoid-rich foods was assessed using a validated, semi-quantitative food frequency questionnaire (FFQ). The FFQ was administered during the preoperative visit (within 7 days prior to surgery) and specifically queried the consumption frequency and portion size of key dietary sources of NAR precursors (e.g., citrus fruits, grapes, tomatoes, and certain herbal teas) over the preceding week. This standardized assessment allowed us to account for and statistically adjust for inter-individual variation in direct dietary exposure to NAR and its glycosidic precursors, ensuring that the observed associations between microbial metabolites and clinical outcomes were independent of recent dietary intake.

##### Histological staining

Ileum tissues (*n* = 8 per group) and small intestine organoids (*n* = 6 per group) were collected and fixed in 4% paraformaldehyde at 4°C overnight, as previously described.^35^ Samples were then sequentially dehydrated, cleared with xylene, and embedded in paraffin. Paraffin-embedded tissue was sectioned at 4 μm thickness and stained with hematoxylin and eosin (H&E) following standard protocols. The severity of ileal injury was scored according to a modified Chiu’s criteria (scale 0–9) by three independent, blinded pathologists based on villus and glandular architecture.^13^ Six randomly selected fields per slide were imaged at 200× or 400× magnification using an Olympus BX63 microscope (Olympus, Japan).

##### Multiplex immunofluorescence staining

Sequential immunofluorescence staining was performed to detect multiple proteins in mouse ileum tissues and small intestine organoids. Briefly, 4-μm paraffin sections underwent antigen retrieval with EDTA buffer (pH 9.0) at 95°C for 20 min, followed by blocking with 10% donkey serum containing 0.3% Triton X-100 at room temperature for 1 h. For ZO-1/Occludin double staining, sections were incubated with rabbit anti-ZO-1 (1:1000), followed by goat anti-rabbit HRP (1:4000) and iFluor 488-tyramide (1:300); after 2% SDS elution (56°C, 15 min), they were stained with rabbit anti-Occludin (1:400), the same secondary, and CY5-tyramide (1:400). For CHAC1/SPRR2A double staining, rabbit anti-CHAC1 (1:400) and HRP-conjugated secondary with tyramide-CY5 (1:400) were applied first, followed by anti-SPRR2A (1:500) and an Alexa Fluor 488-conjugated secondary antibody (1:400). For GPX4/CHAC1/COX-2 triple staining, three sequential rounds were performed: rabbit anti-GPX4 (1:1000) with tyramide-iFluor 488, rabbit anti-CHAC1 (1:400) with tyramide-CY5, and rabbit anti-COX-2 (1:500) with tyramide-CY3. For TLR4/MyD88/SPRR2A triple staining, sections were sequentially incubated with rabbit anti-TLR4 (1:1000) and tyramide-CY3, anti-MyD88 (1:4000) and tyramide-CY5, and rabbit anti-SPRR2A (1:500). For Ki67 single staining, sections were stained with rabbit anti-Ki67 (1:200) and Alexa Fluor 488-conjugated secondary. All primary antibodies were incubated at 4°C overnight; HRP-conjugated secondaries were applied at room temperature for 30 min followed by 10 min tyramide amplification, while direct fluorophore-conjugated secondaries were incubated for 2 h. Nuclei were counterstained with DAPI (10 min), and single-stained and negative controls were included. Fluorescence images were acquired with an Olympus fluorescence microscope (Olympus, Japan), and signal intensities for ZO-1, Occludin, Ki67, SPRR2A, CHAC1, MyD88, TLR4, COX-2, and GPX4 were quantified using ImageJ (NIH, Java 1.8.0) in six randomly selected fields at 200× magnification per sample; for organoid tissues, automated analysis was performed at 400× magnification.

##### Quantitative real-time PCR analysis

Total RNA from small intestine organoids or mouse ileum tissue was extracted using TRIZOL reagent (#15596026, Invitrogen), and reverse transcribed into cDNA with TOYOBO reverse transcriptase (Tokyo, Japan) following the manufacturer’s protocol. Genomic DNA from mouse feces and human stool samples was isolated using a fecal DNA extraction kit (Solarbio, China), and the DNA concentration was standardized to 10 ng/μL. Quantitative real-time PCR was performed on an ABI Q6 Real-Time PCR System using SYBR Green (TOYOBO, Tokyo, Japan). The relative abundance of *B. vulgatus* was calculated using the 2ˆ−ΔΔCT method with 16S rRNA as the internal control, while host gene expression was normalized to 18S rRNA. All primer sequences are listed in Table S3.

##### Surface plasmon resonance (SPR) experiment

Surface plasmon resonance (SPR) was utilized to assess the binding affinities between NAR and recombinant human TLR4 protein (His-tagged, expressed in Sf9 cells; HY-P73586, MedChemExpress), as well as between recombinant human SPRR2A (PA2000-1222, IPODIX) and recombinant human CHAC1 (Strep-tagged; HY-P701945, MedChemExpress). For TLR4 analysis, 3 μL of recombinant protein at 0.35 mg/mL was immobilized on a protein chip and activated with 10 mM NiSO_4_. NAR solutions (2.5, 5, 10, and 20 μM in DPBS) were injected at a flow rate of 30 μL/min. For SPRR2A/CHAC1 analysis, 2 μL of CHAC1 protein at 1.05 mg/mL was immobilized, activated with 10 mM NiSO_4_, and varying concentrations of SPRR2A (0, 62.5, 125, 250, 500, and 1000 nM in DPBS) were introduced at 30 μL/min. Binding signals were recorded using PlexArray HT software (Plexera Bioscience, USA) and analyzed with Origin 9.0 (OriginLab, USA) and BIAevaluation 4.1.1.

##### Assessment of organoid viability and cellular damage

Organoid viability was assessed using the Cell Counting Kit-8 (CCK-8; CK04, Dojindo, Shanghai, China) according to the manufacturer’s instructions. Cellular injury was evaluated by quantifying lactate dehydrogenase (LDH) release in culture supernatants with a commercial LDH assay kit (A020-2-2, Nanjing Jiancheng Bioengineering Institute, Nanjing, China), following the manufacturer’s protocol.

##### Dihydroethidium (DHE) staining for *in situ* reactive oxygen species (ROS)

Frozen intestinal sections (8 μm) were fixed in acetone at −20°C for 10 min, then incubated with 5 μM dihydroethidium (DHE; Thermo D11347) at 37°C for 30 min in the dark. Following three 5-min PBS washes, nuclei were counterstained with DAPI and slides were mounted with fluorescence mounting medium. Confocal images were acquired using a Nikon A1R HD25 microscope with a 20× objective (535 nm excitation, 610 nm emission). Fluorescence intensity, expressed as the percentage area above the sham threshold, was quantified from five randomly selected fields per section using ImageJ.

##### Detection of organoid ROS

To assess reactive oxygen species (ROS) levels in organoids, single-cell suspensions were generated by dissociating organoids with Accutase (Stemcell Cat# 07920) at 37°C for 5 min. Cells were resuspended in PBS at 1 × 10^6^ cells/mL and incubated with 10 μM DCFH-DA for 30 min at 37°C in the dark, then washed twice with PBS to remove excess probe. Flow cytometry was performed on a FACS Calibur (BD Biosciences, USA) with fluorescence detection at 488 nm excitation and 525 nm emission; at least 10,000 viable cells (viability >90% by 7-AAD exclusion) were analyzed per sample using FlowJo software (v10.8, Tree Star).

##### Detection of lipid peroxide by liperfluo staining and flow cytometry

Organoids were enzymatically dissociated into single-cell suspensions using Accutase at 37°C for 5 min, washed with PBS, and stained with 1 μM Liperfluo (Dojindo L248-10) for 30 min at 37°C (5% CO_2_, in the dark). For positive controls, cells were pretreated with 100 μM *tert*-butyl hydroperoxide (tBHP) for 1 h to induce lipid peroxidation. Following staining, cells were resuspended in ice-cold PBS and analyzed using a FACS Calibur flow cytometer (BD Biosciences, USA) with fluorescence detected at 488 nm excitation and 500–550 nm emission. At least 10,000 viable cells were acquired per sample, and data were analyzed using FlowJo software (v10.8, Tree Star).

##### Detection of ferroptosis-related biomarkers in intestinal tissues and organoids

Oxidative stress-related biomarkers in intestinal tissues and organoids were measured using commercial assay kits following the manufacturers’ instructions. Malondialdehyde (MDA) levels in intestinal tissues were determined with the MDA Assay Kit (BC0025, Solarbio, China). Total glutathione (T-GSH) and oxidized glutathione (GSSG) concentrations in both intestinal tissues and organoids were assessed using the T-GSH/GSSG Assay Kit (A061-1-2, Nanjing Jiancheng Bioengineering Institute, Nanjing, China). For Fe^2+^ quantification, Fe^2+^ levels in intestinal tissues were determined with the Fe^2+^ Assay Kit (BC4355, Solarbio, China), while organoid ferrous iron content was measured using the cell ferrous iron colorimetric Assay Kit (E-BC-K881-M, Elabscience, China).

### Quantification and statistical analysis

Statistical analyses were conducted using GraphPad Prism 9.0. Data normality was assessed using the Shapiro-Wilk test, and variance homogeneity with Levene’s test. Data are presented as mean ± SEM unless otherwise specified. Pairwise comparisons were performed using Student’s *t* test for parametric data or the Mann-Whitney U test for non-parametric data. Multi-group comparisons utilized ANOVA followed by Tukey’s post hoc test for homoscedastic data or the Games-Howell test for heteroscedastic data. Survival curves were compared using the log rank test. Correlation analyses were performed using Spearman’s rank correlation coefficient. For clinical data, measurement variables are expressed as mean ± standard deviation and analyzed by *t* test, while categorical data are presented as percentages (%) and compared using the χ^2^ test. A *p* value <0.05 was considered statistically significant; ∗*p* < 0.05, ∗∗*p* < 0.01, ∗∗∗*p* < 0.001. The sample size (n) is specified in each figure legend.

## Supplemental information

Document S1. Figures S1–S7 and Tables S1–S3

Document S2. Article plus supplemental information
