# Supplementary material for: Microbiota-mediated naringenin release limits postoperative acute gastrointestinal injury by suppressing ferroptosis
Source: Cell Rep Med. 2026 Oct 8;7(10):103101. doi: 10.1016/j.xcrm.2026.103101 (PMC13710387; doi:10.1016/j.xcrm.2026.103101)
Supplement: Supplementary file 1 [file mmc1.pdf]

Cell Reports Medicine, Volume *7*

Supplemental information

Microbiota-mediated naringenin release limits postoperative acute gastrointestinal injury by suppressing ferroptosis

Fan Deng, Jingjuan Hu, Yue Min, Sun Xie, Zebin Lin, Qishun Sun, Huamin Liu, Shiying He, Siyuan Feng, Ling Wu, Xiaofeng Chen, Linghong Xu, Zheang Zhang, Wenqi Tang, Zhuang Li, Hongwei Zhou, and Kexuan Liu

Supplemental information

Document S1. Figures S1-S7 and Tables S1-S3.

Supplemental Figure S1

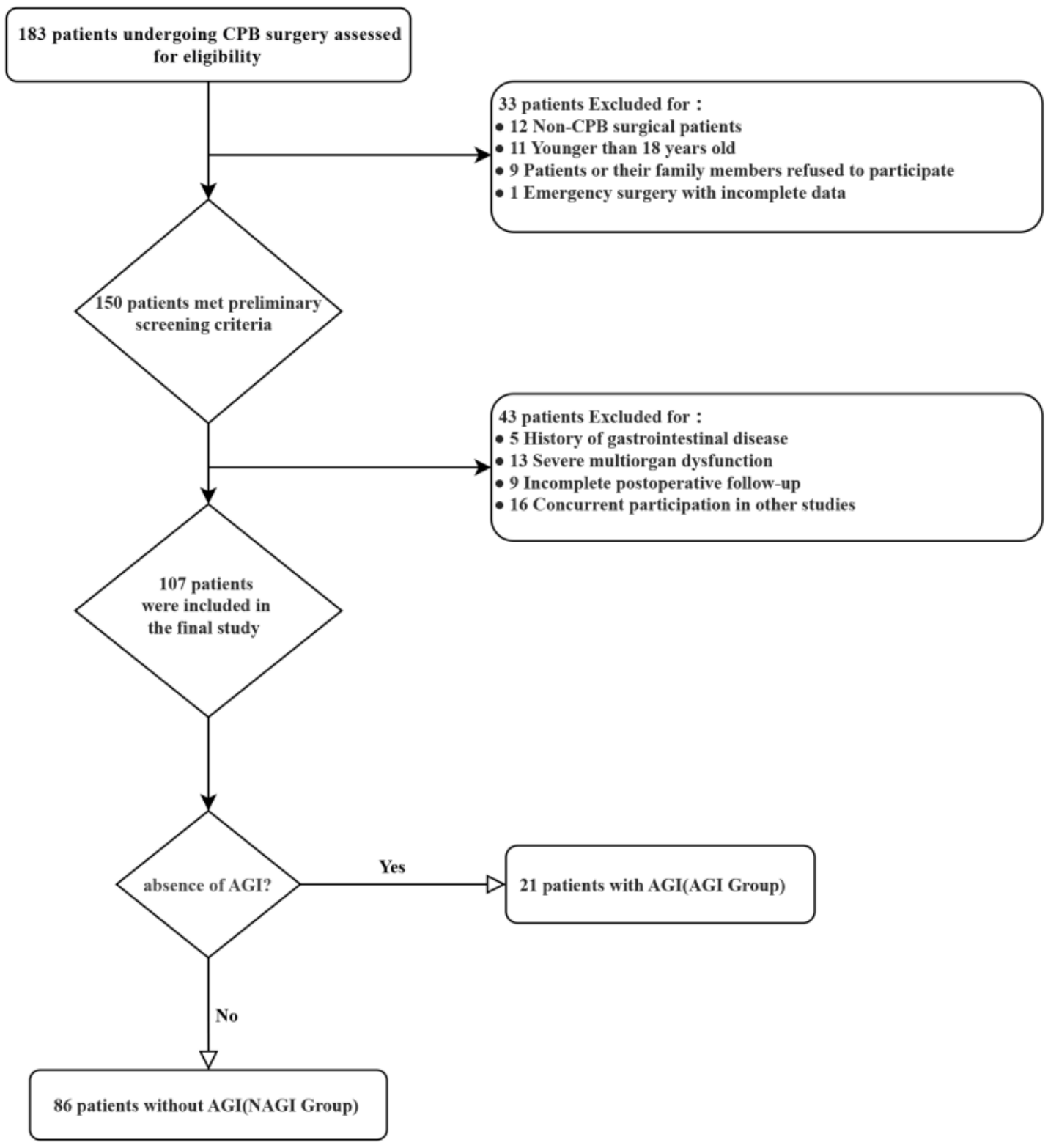

Supplemental Figure S1. Flowchart of CPB patients in derivation cohorts. Related to Figure 1.

Supplemental Figure S2

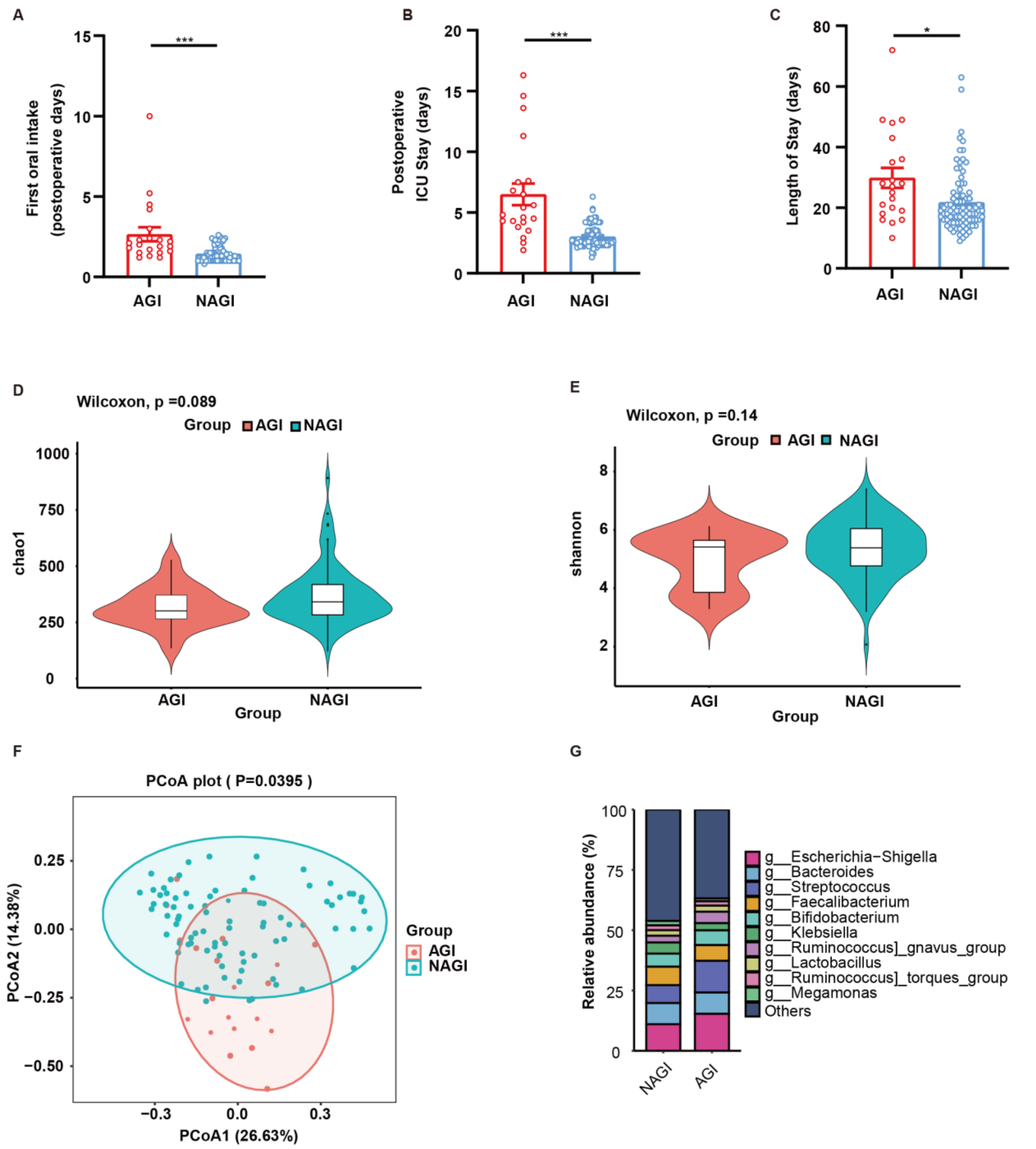

Supplemental Figure S2. Clinical outcomes and gut microbiota profiles in CPB patients. Related to Figures 1 and 2.

(A–C) Clinical outcomes in patients with acute gastrointestinal injury (AGI, n = 21) compared with those without AGI (NAGI, n = 86). The AGI group experienced significantly (A) delayed initiation of oral intake, (B) longer postoperative intensive care unit (ICU) stay, and (C) increased overall length of hospitalisation. (D, E) Alpha diversity indices of preoperative faecal microbiota in AGI versus NAGI patients. (F) PCoA depicting differences in microbial beta diversity between the two groups. (G) Taxonomic composition of gut microbiota at the genus level in AGI and NAGI patients.

Statistical significance was assessed using Student's t-test (A–C) and adonis analysis (F); *$p<0.05$, **$p<0.01$, ***$p<0.001$. Each data point represents an individual biological replicate.

Abbreviations: AGI, acute gastrointestinal injury; ICU, intensive care unit; PCoA, principal coordinate analysis.

Supplemental Figure S3

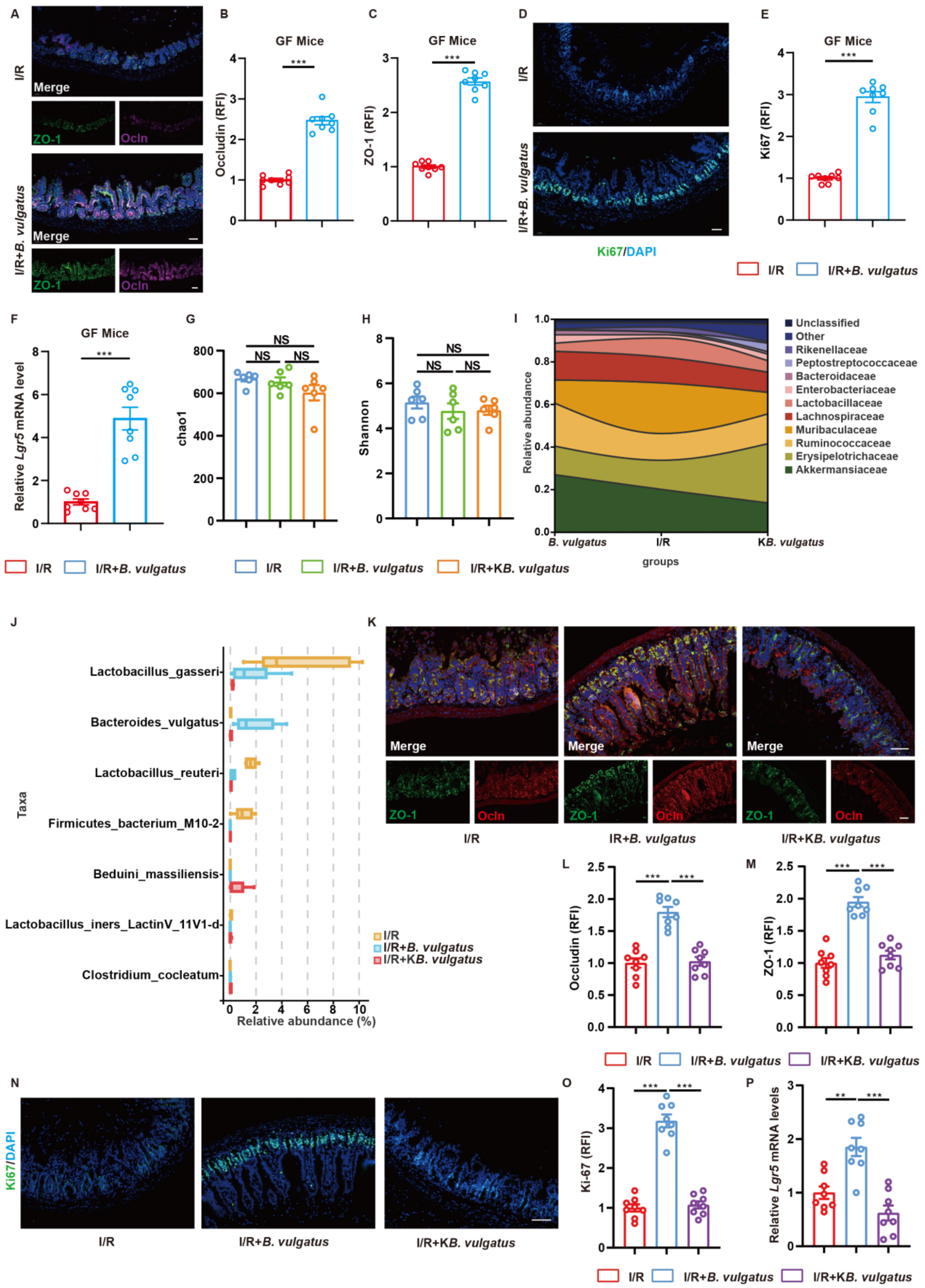

Supplemental Figure S3. Viability-dependent restructuring of gut microbiota by *B. vulgatus* modulates intestinal I/R injury in mice. Related to Figure 3.

(A–E) Immunofluorescence staining and quantitative analysis of ZO-1, Occludin, and Ki67 in intestinal sections from GF mice subjected to I/R or I/R plus *B. vulgatus* treatment; scale bar = 100 µm (n = 8 per group). (F) Relative *Lgr5* mRNA expression in GF mice comparing I/R and I/R + *B. vulgatus* groups (n = 8). (G, H) Alpha diversity analysis (Chao1 and Shannon indices) of gut

microbiota in the indicated groups (n = 6). (I, J) Relative abundance of gut microbiota at the genus (I) and species (J) levels in I/R, I/R + *B. vulgatus*, and I/R + K. *B. vulgatus* groups (n = 6 per group). (K–O) Immunofluorescence staining and quantification of ZO-1, Occludin, and Ki67 in the three groups as above; scale bar = 100 μm (n = 8 per group). (P) Relative *Lgr5* mRNA expression in intestinal tissues across I/R, I/R + *B. vulgatus*, and I/R + K *B. vulgatus* groups (n = 8).

Data are presented as mean ± SEM. Statistical significance was determined using Welch's t-test (A–F) and one-way ANOVA with Tukey's post hoc test (L–P). **p*<0.05, ***p*<0.01, ****p*<0.001. Each data point represents an individual biological replicate. Quantitative measurements were performed in triplicate.

Abbreviations: GUS, β-glucuronidase; GF, germ-free; K. *B. vulgatus*, heat-killed *Bacteroides vulgatus*; NS, not significant; RFI, relative fluorescence intensity.

Supplemental Figure S4

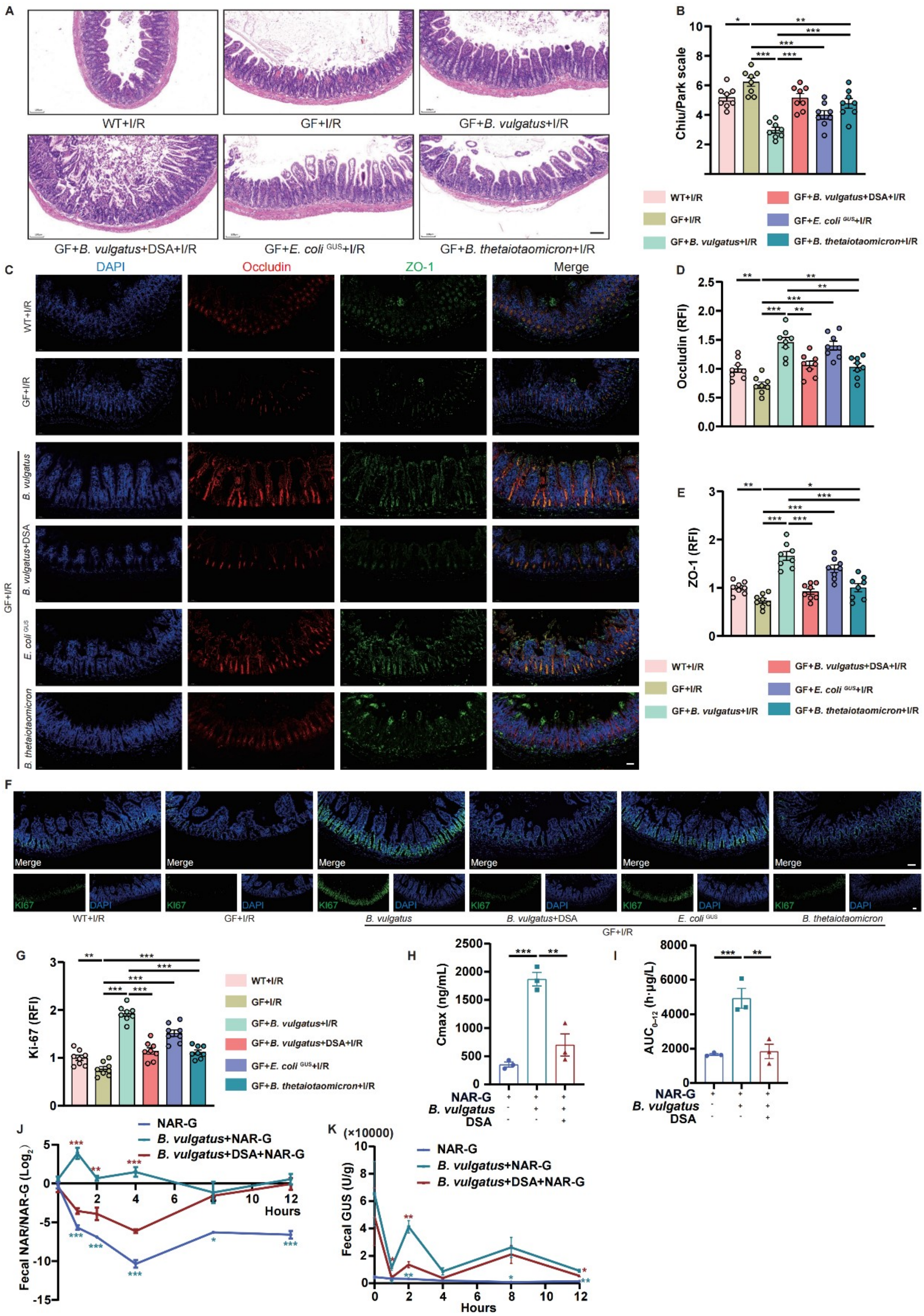

Supplemental Figure S4. GUS-dependence of *B. vulgatus*-associated protection against intestinal I/R injury. Related to Figure 3.
(A, B) Representative H&E staining (A) and quantification of pathological damage scores (B) in intestinal tissues (scale bar: 100 μm; n = 8 per group). (C) Representative immunofluorescence images

of the ileal mucosa stained for the tight junction proteins ZO-1 and Occludin. Scale bar: 50 μm. (D, E) Quantitative analysis of ZO-1 (D) and Occludin (E) integrated fluorescence intensity in the mucosal epithelium (n = 8 mice per group). (F) Representative images of Ki67 immunofluorescence in intestinal crypts. Scale bar: 50 μm. (G) Quantification of Ki67-positive cells per crypt (n = 8 mice per group). (H) Peak fecal NAR concentration (n = 3 mice per group). (I) Area under the fecal concentration–time curve ($AUC_{0–12}$) of NAR (n = 3 mice per group). (J) Ratio of free NAR to NAR-G in feces (n = 4 mice per group). (K) Temporal changes in fecal GUS activity (n = 4 mice per group). For pharmacokinetic studies (H-K), corresponding colonized GF mice were gavaged with NAR-G or vehicle.

Data are presented as mean ± SEM. Statistical analysis was performed by one-way ANOVA with Tukey's post hoc test for multiple comparisons. *$p < 0.05$, **$p < 0.01$, ***$p < 0.001$. Each data point represents an individual biological replicate. Quantitative measurements were performed in triplicate. The asterisks of different colors (*, **, ***) in the figure are used to indicate the statistical significance of intergroup comparisons, with the color of the asterisks matching the color of the corresponding group.

Supplemental Figure S5

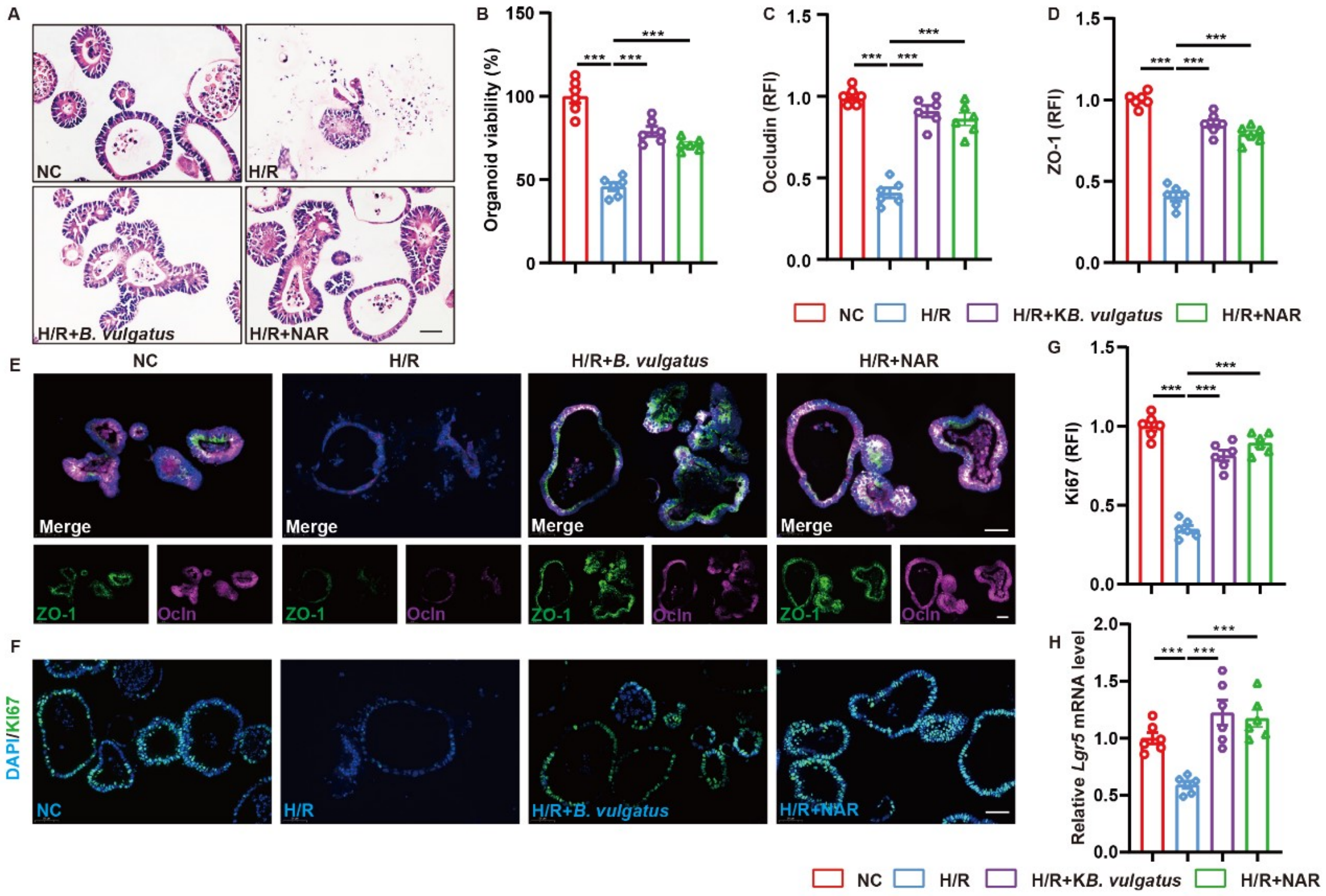

Supplemental Figure S5. *B. vulgatus* and NAR protect intestinal organoids from hypoxia/reoxygenation (H/R) injury. Related to Figure 3.
(A) Representative H&E staining of intestinal organoids (scale bar: 50 μm; n = 6 per group). (B) Quantification of intestinal organoid viability across negative control (NC), hypoxia/reoxygenation (H/R), H/R + *B. vulgatus*, and H/R + NAR groups (n = 6 per group). (C–E) Immunofluorescence staining and quantitative analysis of ZO-1 and Occludin expression in intestinal organoids (scale bar: 50 μm; n = 6 per group). (F, G) Immunofluorescence staining and quantification of Ki67-positive cells in organoids (scale bar: 50 μm; n = 6 per group). (H) Relative mRNA expression of *Lgr5* in intestinal organoids (n = 6 per group). Data are presented as mean ± SEM. Statistical analysis was performed by one-way ANOVA with Tukey's post hoc test for multiple comparisons. *$p < 0.05$, **$p < 0.01$, ***$p < 0.001$. Each data point represents an individual biological replicate. Quantitative measurements were performed in triplicate.

Supplemental Figure S6

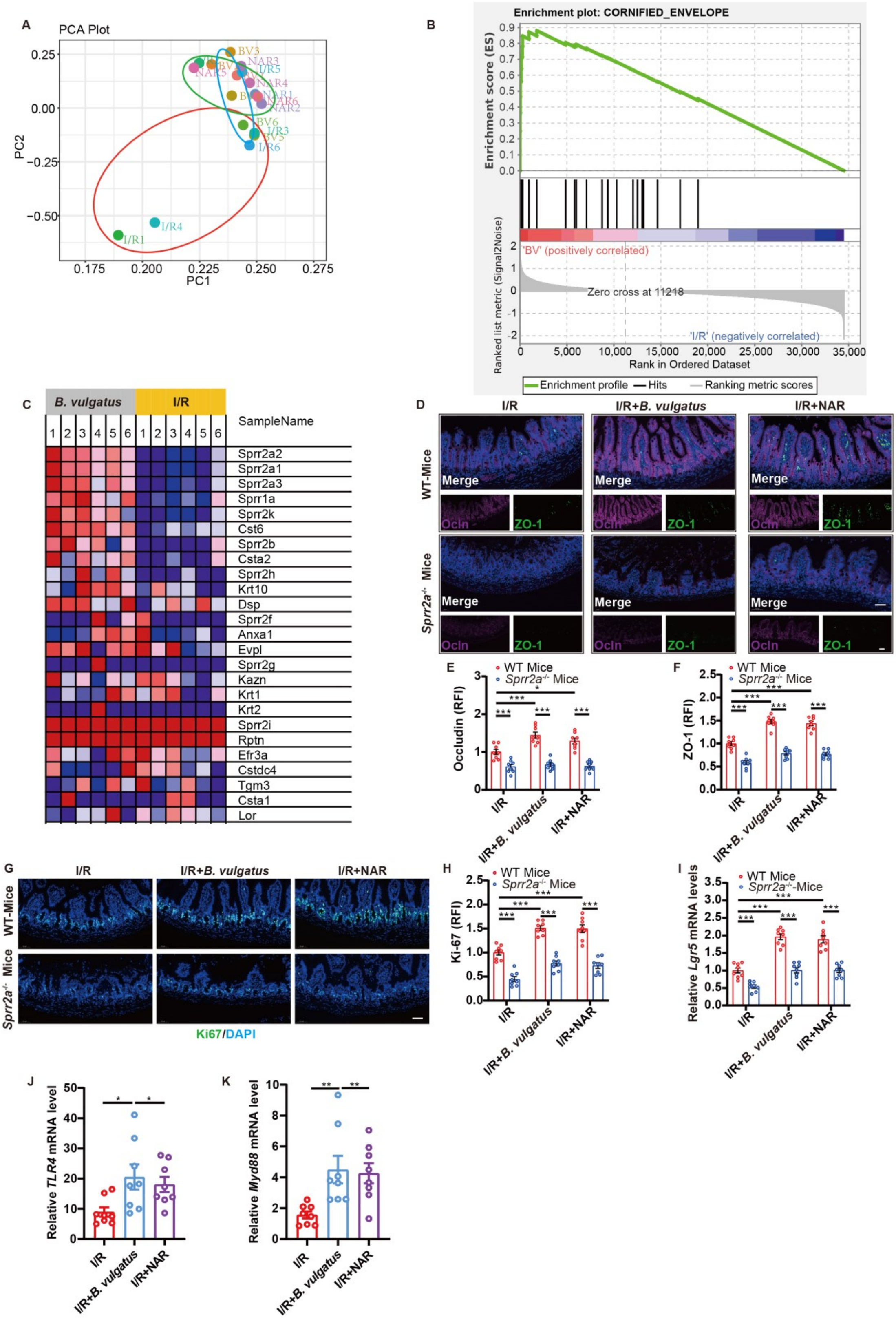

Supplemental Figure S6. *B. vulgatus* activates TLR4/MyD88-SPRR2A signaling to suppress CHAC1-mediated barrier dysfunction. Related to Figure 4.
(A) PCA of transcriptomic profiles from intestinal tissues of mice in the I/R, I/R + Bacteroides vulgatus, and I/R + NAR groups (n = 6 per group). (B, C) Gene set enrichment analysis (GSEA) of

intestinal transcriptomes illustrating Sprr2a enrichment in *B. vulgatus*-treated I/R mice. (D–F) Immunofluorescence staining and quantitative analysis of ZO-1 and Occludin in intestinal tissues from WT and *Sprr2a*$^{-/-}$ mice following I/R, I/R + *B. vulgatus*, or I/R + NAR treatment; scale bar = 100 µm (n = 8 per group). (G, H) Immunofluorescence staining and quantification of Ki67 in intestinal tissues from WT and *Sprr2a*$^{-/-}$ mice; scale bar = 100 µm (n = 8 per group). (I) Relative *Lgr5* mRNA expression in WT or *Sprr2a*$^{-/-}$ mice (n = 8 per group). (J, K) Relative *Tlr4* and *Myd88* mRNA expression in WT mice across the Sham, I/R, I/R + *B. vulgatus*, and I/R + NAR groups (n = 8 per group).
Data are presented as mean ± SEM. Statistical significance was assessed by two-way ANOVA with Tukey's post hoc test (E–I) and one-way ANOVA with Tukey's post hoc test (J, K). **p*<0.05, ***p*<0.01, ****p*<0.001. Each data point represents an individual biological replicate. Quantitative measurements were performed in triplicate.
Abbreviations: PCA, principal component analysis; WT, wild-type; RFI, relative fluorescence intensity.

Supplemental Figure S7

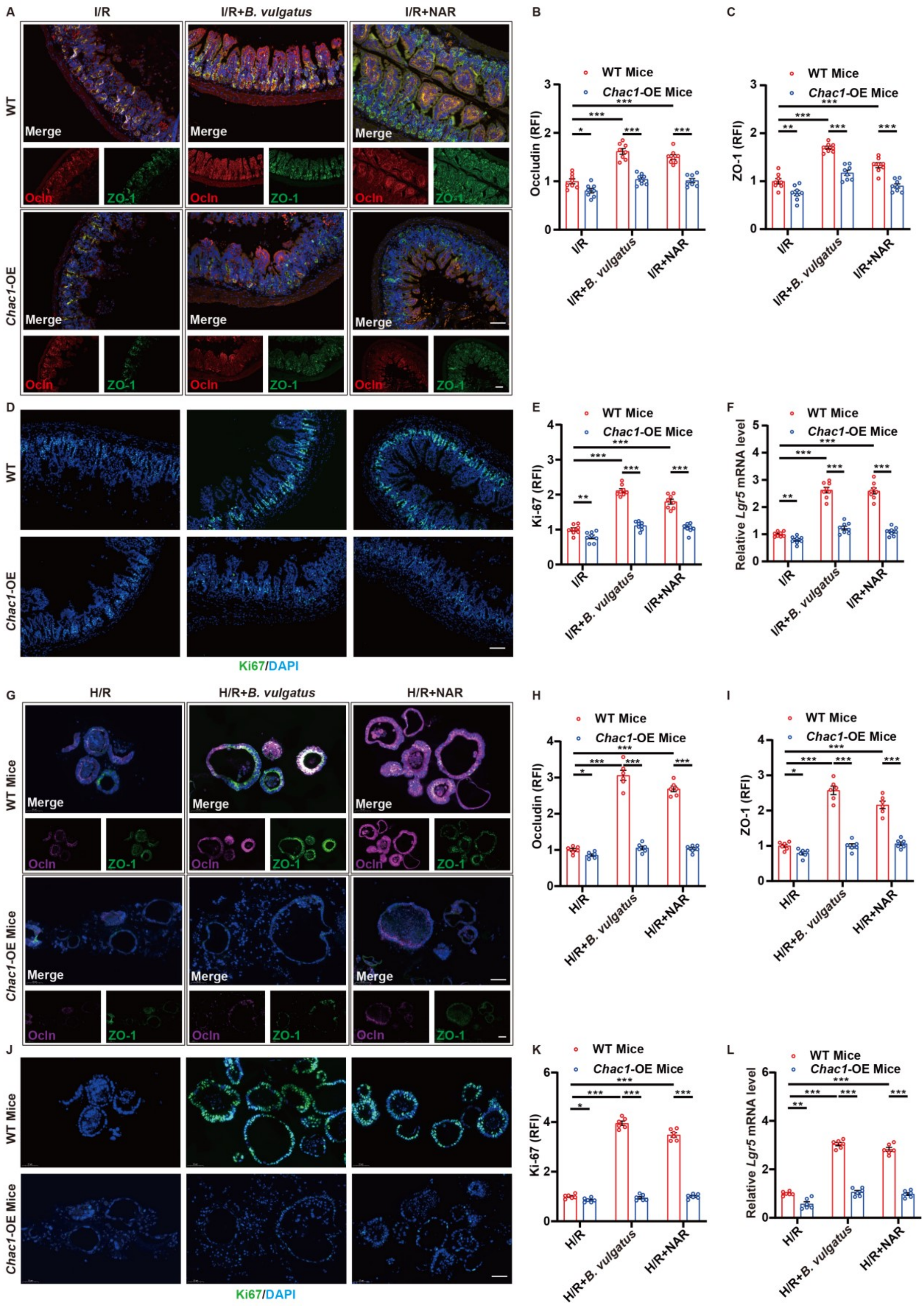

Supplemental Figure S7. CHAC1 overexpression abrogates the protective effects of *B. vulgatus* and NAR on the intestinal barrier. Related to Figures 5 and 6.
(A–C) Multiplex immunofluorescence staining of ZO-1 and Occludin (scale bar = 100 μm; n = 8) and quantitative analysis of ZO-1 and Occludin expression in intestinal tissues derived from WT and Chac1-overexpressing (Chac1-OE) mice (n = 8 per group). (D, E) Immunofluorescence staining and quantification of Ki67 in intestinal tissues from WT and Chac1-OE mice (scale bar = 100 μm; n = 8 per

group). (F) Relative mRNA expression of *Lgr5* in intestinal tissues (n = 8 per group). (G) Multiplex immunofluorescence staining of ZO-1 and Occludin in intestinal organoids from WT and Chac1-OE mice (scale bar = 50 μm; n = 6 per group). (H, I) Quantitative analysis of ZO-1 and Occludin in organoids (n = 6 per group). (J, K) Immunofluorescence staining and quantification of Ki67 in organoids (scale bar = 50 μm; n = 6 per group). (L) Relative mRNA expression of *Lgr5* in organoids (n = 6 per group).

Data are presented as mean ± SEM. Statistical analysis was performed using two-way ANOVA with Tukey's post hoc test. *$p<0.05$, **$p<0.01$, ***$p<0.001$. Each data point represents an individual biological replicate. Quantitative measurements were performed in triplicate.

Abbreviation: RFI, relative fluorescence intensity.

Supplemental Table S1. The baseline characteristics and intraoperative parameters of CPB patients in modeling cohorts. Related to Figure 1.

Supplementary Table S1. Baseline characteristics and perioperative details of CPB patients in the modeling cohort.

Variable	Overall, N = 107[1]	AGI, N = 21 (19.6%)[1]	NAGI, N = 86 (80.4%)[1]	Statistics[2]	P-value[2]
**Demographics**					
Age (years)	56.00 [48.50, 62.00]	53.00 [43.00, 61.00]	56.00 [50.00, 62.00]	796	0.403
Female, n (%)	62 (57.94%)	16 (76.19%)	46 (53.49%)	2.7	0.1
BMI (Kg/m²)	23.55 (3.42)	24.07 (3.71)	23.42 (3.35)	0.77	0.441
Preoperative Medications, n (%)					
Statins	88 (82.2%)	18 (85.7%)	70 (81.4%)	0.1	0.755
Beta-blockers	73 (68.2%)	15 (71.4%)	58 (67.4%)	0.07	0.799
Anti-platelets	67 (62.6%)	12 (57.1%)	55 (64.0%)	0.24	0.627
Proton Pump Inhibitors (PPI)	20 (18.7%)	7 (33.3%)	13 (15.1%)	3.78	0.052
**Surgery & Intraoperative Details**					
CPB time (mins)	138.00 [103.00, 175.00]	134.00 [125.00, 187.00]	139.50 [98.50, 172.50]	1,057.00	0.229
Operation time (mins)	273.00 [240.00, 320.00]	273.00 [254.00, 308.00]	273.50 [240.00, 341.00]	914	0.934
Anesthesia time (mins)	315.00 [286.50, 370.00]	311.00 [305.00, 370.00]	315.00 [280.00, 370.00]	873	0.816
Intraoperative Vasoactive agents, n (%)	107 (100.0%)	21 (100.0%)	86 (100.0%)	N/A	1
Intraoperative Corticosteroids, n (%)	39 (36.4%)	15 (71.4%)	24 (27.9%)	13.86	**<0.001**
**Postoperative Management & Support**					
Any Postoperative Antibiotics, n (%)	107 (100.0%)	21 (100.0%)	86 (100.0%)	N/A	1
Upgraded to Broad-spectrum Antibiotics, n (%)	28 (26.2%)	15 (71.4%)	13 (15.1%)	28.97	**<0.001**
Postoperative Vasoactive agents, n (%)	39 (36.4%)	18 (85.7%)	21 (24.4%)	27.6	**<0.001**
Primary Nutritional Support, n (%)				66.29	**<0.001**
Enteral Nutrition (EN)	71 (66.4%)	3 (14.3%)	68 (79.1%)		
Parenteral Nutrition (PN)	27 (25.2%)	17 (81.0%)	10 (11.6%)		
Both	9 (8.4%)	1 (4.8%)	8 (9.3%)		
**Outcomes & Complications**					

Days of Hospitalization (days)	19.00 [16.00, 28.00]	27.00 [18.50, 39.50]	19.00 [15.50, 24.50]	1,500.20	0.01
ICU Stay (days)	3.00 [1.00, 5.00]	7.50 [5.00, 12.00]	2.00 [1.00, 4.00]	1,520.00	**<0.001**
Onset Duration after CPB (days)		2.5 [2.0, 3.0]			
Acute Kidney Injury (AKI), n (%)	19 (17.8%)	11 (52.4%)	8 (9.3%)	21.63	**<0.001**
Prolonged Mechanical Ventilation (>48h), n (%)	17 (15.9%)	10 (47.6%)	7 (8.1%)	20.41	**<0.001**
3-Month Mortality, n (%)	8 (7.48%)	5 (23.81%)	3 (3.49%)	N/A[3]	**0.007**

Footnotes:

[1] Data presented as Median [IQR]; n (%); or Mean (SD).

[2] P-values were derived from Wilcoxon rank sum test (continuous non-parametric), Pearson's Chi-squared test, Two Sample t-test (continuous parametric), or Fisher's exact test, as appropriate. The statistic value (Z, χ^2, t, or N/A) is provided.

[3] P-value for 3-Month Mortality derived from Fisher's exact test due to small expected counts.

Abbreviations: AGI, acute gastrointestinal injury; NAGI, non-acute gastrointestinal injury; CPB, cardiopulmonary bypass; BMI, body mass index; ICU, intensive care unit; AKI, acute kidney injury.

Supplemental Table S2. Analysis of potential confounding factors relating to NAR sources and metabolism in CPB modeling cohort. Related to Figure 1.

CPB					
**Variable**	**Overall, N = 107**[1]	**AGI N = 21 (20%)**[1]	**NAGI N = 86 (80%)**[1]	**statistics**	***P*-value**[2]
**Natural dietary sources**				0.24	0.623
No	88 (82.24%)	16 (76.19%)	72 (83.72%)		
Yes	19 (17.76%)	5 (23.81%)	14 (16.28%)		
**Dietary supplements**					0.484
No	104 (97.20%)	20 (95.24%)	84 (97.67%)		
Yes	3 (2.80%)	1 (4.76%)	2 (2.33%)		
**CYP3A4 UGT inhibitors**					
No	107 (100.00%)	21 (100.00%)	86 (100.00%)		
**Disease status**				0.97	0.325
No	87 (81.31%)	15 (71.43%)	72 (83.72%)		
Yes	20 (18.69%)	6 (28.57%)	14 (16.28%)		

[1]Median [IQR]; n (%); Mean (SD)

[2]Wilcoxon rank sum test; Pearson's Chi-squared test; Two Sample t-test; Fisher's exact test

Supplemental Table S3. Quantitative RT-PCR primer sequence. Related to STAR Methods section.

Gene	Forward primer (5'-3')	Reverse primer (5'-3')
16S	GTGSTGCAYGGYTGTCGTCA	ACGTCRTCCMCACCTTCCTC
18S	CGATCCGAGGGCCTCACTA	AGTCCCTGCCCTTTGTACACA
Lgr5	CCTACTCGAAGACTTACCCAGT	GCATTGGGGTGAATGATAGCA
Sprr2a	GCCTTGTCGTCCTGTCATGT	GGCATTGCTCATAGCACACTAC
TLR4	TGTCATCAGGGACTTTGCTG	GGACTCTGATCATGGCACTG
Myd88	TCATGTTCTCCATACCCTTGGT	AAACTGCGAGTGGGGTCAG
B. vulgatus	ACGATGAATACTCGCTGTTTGC	CATGTTCCTCCGCTTGTGC
